# Hypercapnia Causes Injury of the Cerebral Cortex and Cognitive Deficits in Newborn Piglets

**DOI:** 10.1523/ENEURO.0268-23.2023

**Published:** 2024-03-01

**Authors:** Karen Fritz, Georgios Sanidas, Rodolfo Cardenas, Javid Ghaemmaghami, Chad Byrd, Gabriele Simonti, Adriana Valenzuela, Ignacio Valencia, Maria Delivoria-Papadopoulos, Vittorio Gallo, Ioannis Koutroulis, Terry Dean, Panagiotis Kratimenos

**Affiliations:** ^1^Drexel University College of Medicine, Philadelphia, Pennsylvania 19104; ^2^Department of Pediatrics, St. Christopher’s Hospital for Children, Philadelphia, Pennsylvania 19134; ^3^Center for Neuroscience Research, Children’s National Research Institute, Washington, DC 20010; ^4^Department of Pediatrics, Children’s National Hospital, Washington, DC 20010; ^5^Seattle Children’s Research Institute, Seattle, Washington 98101; ^6^The George Washington University School of Medicine and Health Sciences, Washington, DC 20052

**Keywords:** cortex, hypercapnia, hypercarbia, neonatal, permissive, piglet

## Abstract

In critically ill newborns, exposure to hypercapnia (HC) is common and often accepted in neonatal intensive care units to prevent severe lung injury. However, as a “safe” range of arterial partial pressure of carbon dioxide levels in neonates has not been established, the potential impact of HC on the neurodevelopmental outcomes in these newborns remains a matter of concern. Here, in a newborn Yorkshire piglet model of either sex, we show that acute exposure to HC induced persistent cortical neuronal injury, associated cognitive and learning deficits, and long-term suppression of cortical electroencephalogram frequencies. HC induced a transient energy failure in cortical neurons, a persistent dysregulation of calcium-dependent proapoptotic signaling in the cerebral cortex, and activation of the apoptotic cascade, leading to nuclear deoxyribonucleic acid fragmentation. While neither 1 h of HC nor the rapid normalization of HC was associated with changes in cortical bioenergetics, rapid resuscitation resulted in a delayed onset of synaptosomal membrane lipid peroxidation, suggesting a dissociation between energy failure and the occurrence of synaptosomal lipid peroxidation. Even short durations of HC triggered biochemical responses at the subcellular level of the cortical neurons resulting in altered cortical activity and impaired neurobehavior. The deleterious effects of HC on the developing brain should be carefully considered as crucial elements of clinical decisions in the neonatal intensive care unit.

## Significance Statement

Hospitalized critically ill neonates commonly experience hypercapnia (HC) as part of common practice in neonatal intensive care, as ventilated newborns are subjected to “permissive HC” to reduce lung injury associated with positive pressure ventilation. To answer the clinically important question of how “safe” that practice is, the present study provides new insights into the complex effects of HC on neuronal processes, with significant ramifications for ongoing neurodevelopment.

## Introduction

Hospitalized critically ill neonates commonly experience hypercapnia (HC) as part of a widespread practice in neonatal intensive care, as ventilated newborns are subjected to “permissive HC” to reduce lung injury associated with positive pressure ventilation (Wong et al., 2022). However, far less attention has been paid to the effects of supraphysiologic partial pressure of carbon dioxide (PaCO_2_) on neonatal brains. Clinical studies suggest that permissive HC is “safe” (Mariani et al., 1999; Woodgate and Davies, 2001; Varughese et al., 2002; Rojas-Reyes et al., 2012; Ozawa et al., 2022), although the majority do not address long-term outcomes. While it has not been associated with the development of gross neurologic insults (e.g., intraventricular hemorrhage) in more recent studies (Volpe, 1981; Szymonowicz et al., 1984; Akhter et al., 2001; Wong et al., 2022), HC has been associated with impaired brainstem auditory-evoked responses (Friss et al., 1994) and poor neurologic outcomes (Pryds et al., 1990; Muller et al., 1997), suggestive of an under-recognized HC-induced brain injury. However, the impact of HC on different brain regions and the exact cellular and molecular mechanisms underlying the consequences of HC on brain development are yet to be elucidated. Unsurprisingly, an optimal range and length of exposure for PaCO_2_ in newborns remains to be established (Woodgate and Davies, 2001; Fabres et al., 2007; Thome et al., 2015; Waitz et al., 2016; Lampe et al., 2020).

As it is challenging to account for variable patient comorbidities and illness severities present in human clinical data, animal models of HC provide an opportunity to better resolve a relationship between neonatal HC and neurologic injury. The structure and function of the piglet brain closely resemble that of a human term brain, and pigs are increasingly utilized for neurological research on human conditions (Gieling et al., 2011). Using a newborn piglet model of HC, it has been previously demonstrated that 6 h of isolated HC acutely precipitates cellular energy failure and dysregulation of calcium-dependent intracellular processes that may lead to proapoptotic signaling in cortical neurons (Fritz et al., 2005; Fritz and Delivoria-Papadopoulos, 2006). However, most critically ill newborns undergo resuscitation and recover rather than experiencing prolonged HC. The effects of resuscitation and recovery on brain function following HC remain largely unknown. Therefore, in this study, we took a more translational approach to investigate the impact of HC within clinically related contexts. In our piglet model, we used a 3 h duration of HC, to replicate short-term neonatal clinical scenarios. Additionally, we explored the effects of 1 h HC exposure with and without rapid CO_2_ correction, simulating acute respiratory failure scenarios in neonatal intensive care units (NICUs), to evaluate the impact of rapid resuscitation. Expanding on our previous research, we initially investigated whether the HC-induced biochemical changes in the cortex correlated with cognitive and executive deficits, by using a novel behavioral assessment protocol. Subsequently, we explored whether HC had a direct and acute effect on the cerebral cortex by monitoring electroencephalogram (EEG) alterations. Considering the findings from cortical neurobehavioral tests and observed electrographic changes following HC, we focused our biochemical analysis on neurons of the cerebral cortex. Our assessments demonstrated alignment of these cortical processes, strongly suggesting that HC indeed affects the cerebral cortex.

## Materials and Methods

### Ethics approval

All animal procedures were performed in accordance with the regulations of the Institutional Animal Care and Use Committee of the Drexel University College of Medicine and/or MCP Hahnemann University College of Medicine (IACUC #02264, #02747, and #02882-01).

### Experimental protocols

The following three main groups of newborn Yorkshire piglets of either sex were studied: (1) spontaneous 7 d recovery of noninstrumented piglets exposed to 3 h of severe HC (PaCO_2_, 80 mmHg) in a chamber followed by 7 d of recovery in room air; (2) rapid resuscitation, ventilated piglets exposed to moderate (PaCO_2_, 65 mmHg) or severe HC (PaCO_2_, 80 mmHg) for 1 h followed by resuscitation and 1 h of normocapnic ventilation; and (3) acute ventilated piglets exposed to either moderate or severe HC for 6 h. We compared each of the groups with a corresponding sham group of normocapnia (NC) piglets (Extended Data Fig. 1-1). Baseline physiologic data were comparable within the groups. We utilized blood gas measurements to validate the establishment of moderate or severe HC and related acidosis in the corresponding groups of piglets. All piglets were normoxic (Tables 1,2). PaCO_2_ levels were chosen to mimic levels chosen for permissive HC in NICUs. Prior to being studied, the piglets were housed in a group pen in the animal facility.

**Table 1.** Arterial blood gases of newborn piglets at the end of 3 h of acute chamber HC (13% FiCO_2_)

Groups	pH (a)	PaCO_2_ (mmHg)	PaO_2_ (mmHg)
Acute HC *n* = 6	7.30 ± 0.03	73 ± 9	80 ± 27

**Table 2.** Effect of moderate and severe HC and resuscitation on neuronal function in newborn piglets physiologic data

Groups	pH (a)	PaCO_2_ (mmHg)	PaO_2_ (mmHg)	Mean BP (mmHg)	Heart rate (bpm)
NC (PaCO_2_ 40 mmHg 1 h) *n* = 6 (PaCO_2_ 40 mmHg 2 h) *n *= 7	7.47 ± 0.05	42 ± 3	95 ± 14	85 ± 10	230 ± 27
Hypercapnic 65 Study period (PaCO_2_ 65 mmHg 1 h) *n* = 6	7.27 ± 0.04*	66 ± 3*	102 ± 10	81 ± 12	221 ± 20
Hypercapnic 65-rec Recovery period (PaCO_2_ 65 mmHg 1 h then PaCO_2_ 40 1 h) *n* = 5	7.49 ± 0.02	50 ± 19	87 ± 22	99 ± 9	251 ± 22
Hypercapnic 80 Study period (PaCO_2_ 80 mmHg 1 h) *n* = 8	7.21 ± 0.06**	82 ± 8**	99 ± 14	79 ± 13	219 ± 25
Hypercapnic 80-rec Recovery period (PaCO_2_ 80 mmHg 1 h then PaCO_2_ 40 1 h) *n* = 4	7.47 ± 0.05	42 ± 4	101 ± 12	94 ± 10	205 ± 33

*p < 0.05, **p < 0.01.

#### Spontaneous 7 d recovery

Thirty-five spontaneously breathing, nonventilated, noninstrumented, sedated piglets were placed in an animal chamber for 3 h to determine the effects of HC on learning, behavior, and EEGs and the persistence of tissue alterations at 7 d post HC. For this group, we opted for the 3 h duration of HC for two primary reasons. Firstly, 3 h of HC more closely resembles the conditions encountered in a clinical NICU scenario such as acute deterioration in mechanically ventilated patients, endotracheal tube dislodgement, postoperative ventilatory adjustments, and other similar scenarios within the 3 h range. Given this clinical relevance, it would be beneficial to determine if a shorter duration of HC would result in brain injury or if longer exposure was necessary. Additionally, we considered the need to ensure the piglet's survival for behavioral assessments 7 d following HC exposure. Prolonged hypercapnic exposure would have required additional interventions such as intravenous (IV) fluids, dextrose administration, and increased sedation. This added instrumentation could increase the risk of infection (Fritz et al., 2005). Three- to 5-d-old newborn piglets were sedated with 0.8 cc intramuscular dose (20 mg/kg, i.m.) of Nembutal (sodium pentobarbital 50 mg/1 cc solution) and placed into a controlled animal chamber for 3 h. Chamber oxygen saturations of 21–22% and desired CO_2_ (13%) were continuously monitored and maintained at desired ranges with added CO_2_, O_2_, and airflow. Piglet and chamber temperature were continuously monitored via air and skin sensors. Piglet skin temperatures were kept at 38°C by heating blankets and heat lamps, and chamber temperatures were at 40°C. Piglet respiratory rates were recorded every 15 min. Additional intramuscular doses of 0.2 cc Nembutal were given for movement. Due to the piglet's intermittent movement, skin pulse oximetry and respiratory probes were not able to be used. To limit infections, no IV or arterial lines were inserted. One femoral arterial blood gas was performed at the end of the experimental period under sterile conditions on the HC piglets while in the sealed chamber (Extended Data Fig. 1-1a). These data are presented in Table 1. Piglets were divided into one of four groups—acute severe HC, 80 3 h (where the piglets were exposed to 13% CO_2_ and 21% O_2_ for 3 h and then killed, *n* = 6); acute NC, 40 3 h (piglets exposed to 21% O_2_ for 3 h and then killed, *n* = 7); recovery HC (piglets allowed to recover in room air for 7 d after exposure to 13% CO_2_ and 21% O_2_ for 3 h, 80 7 d, *n* = 13); or recovery NC (piglets allowed to recover for 7 d after exposure to 21% O_2_ for 3 h, 40 7 d, *n* = 9). For the HC recovery piglets, the additional CO_2_ was turned off, and the chamber was opened. The recovery piglets were allowed to spontaneously wake up while being continuously monitored until they could walk and feed on their own. They were then returned to their original group pen for 7 d where they underwent daily behavioral and cognitive testing. Prior to sacrifice, the piglets were given a dose of intraperitoneal Nembutal 3.0 cc (75 mg/kg/dose) and decapitated. There was no difference in Nembutal dosing between the NC and HC groups. The cerebral cortices were rapidly removed within seconds and divided into two groups. One group was placed in a buffer for cellular fraction isolation, while the other was immediately frozen in liquid nitrogen and stored at −80°C for later analysis of adenosine triphosphate (ATP), phosphocreatine (PCr), and lipid peroxidation products. Given the size of the piglet brain, extraction was specifically limited to the cerebellar cortex with careful examination to exclude any cerebellum or hindbrain tissue.

#### Rapid resuscitation

We studied thirty-five anesthetized, intubated, and ventilated piglets divided into six groups, three nonresuscitated, and three resuscitated. To closer mimic the common clinical scenario of an infant with HC, the protocol utilized piglets exposed to only 1 h of HC followed by rapid resolution of the HC within 15 min. Anesthesia was induced with 4% isoflurane and maintained with 0.8% isoflurane. Lidocaine 1% was injected locally for the performance of a tracheostomy and femoral arterial line insertion. A peripheral or femoral cut-down IV line was inserted, and IV fentanyl (10 µg/kg every hour and as needed) and pavulon (0.1 mg/kg every 1–2 h and as needed) were given. The animals were placed on a pressure ventilator using 75% nitrous oxide and 25% oxygen. A femoral aortic catheter was inserted to continuously monitor heart rate and blood pressure as well as to obtain blood samples for analysis. Arterial blood pH, PaO_2_, PaCO_2_, heart rate, oxygen saturation, and blood pressure were continuously monitored and recorded every 5–15 min in all animals. Physiologic data for these piglets are reported in Table 2 and demonstrate that both the 65 1 h and the 80 1 h groups had significantly lower pH levels and higher PaCO_2_ levels than the NC piglets. All other physiologic parameters were not different between the groups. Temperature was maintained with a warming blanket and warming lights and was continuously monitored. After stabilization, the piglets were divided into six groups. The nonresuscitated piglets consisted of those ventilated with CO_2_ added into the respiratory circuit to maintain a PaCO_2_ of 65 mmHg and PaO_2_ 80–100 mmHg (65 1 h group, *n* = 6), those ventilated with added CO_2_ to maintain a PaCO_2_ of approximately 80 ± 2 mmHg and a PaO_2_ 80–100 mmHg (80 1 h group, *n* = 8), and those ventilated with no added CO_2_ for 1 h with a PaCO_2_ of 40 ± 2 mmHg and a PaO_2_ 80–100 mmHg (NC group, *n* = 6). The resuscitated piglets included those resuscitated after having a PaCO_2_ of 65 mmHg for 1 h (65 1 h + resuscitation, *n* = 5), those resuscitated after having a PaCO_2_ of 80 mmHg for 1 h (80 1 h + resuscitation, *n* = 4), and those continuing normoxic NC ventilation after having a PaCO_2_ of 40 mmHg for 1 h (40 1 h + resuscitation, *n* = 7). Resuscitation occurred by discontinuing the CO_2_ added to the ventilator circuit and allowing normoxic (PaO_2_ 80–100 mmHg) NC (PaCO_2_ 38–42 mmHg) ventilation for an additional hour. The ventilator settings were not changed during resuscitation. Most piglets had the normalization of PaCO_2_ within 10–15 min after the discontinuation of added CO_2_. At the end of the experimental period, the piglets were given an additional dose of fentanyl IV (10 µg/kg) and were decapitated. Cerebral cortices were rapidly removed within seconds as the recovery piglet brains above.

#### Acute ventilated

Six hours of HC: We studied twenty-one anesthetized and intubated piglets divided into three subgroups. Piglets were prepared as per the methods under the “Rapid resuscitation” section above. After stabilization, the piglets were divided into three groups—two HC and one NC. The HC piglets consisted of two groups—those ventilated with CO_2_ and O_2_ added into the respiratory circuit to maintain a PaCO_2_ of 65 ± 2 mmHg and PaO_2_ 80–100 mmHg (65 6 h, *n* = 7) for 6 h and those ventilated with added CO_2_ to maintain a PaCO_2_ of approximately 80 ± 2 mmHg and a PaO_2_ of 80–100 mmHg for 6 h (80 6 h, *n* = 7). The NC piglets were ventilated for 6 h with a PaCO_2_ of 40 ± 2 mmHg and a PaO_2_ of 80–100 mmHg (40 6 h, *n* = 7). At the end of the experimental period, the piglets were given an additional dose of fentanyl IV (10 µg/kg) and decapitated. Brain tissue was processed as under the recovery group. The physiologic data for the 6 h acutely ventilated piglets were previously published (Fritz et al., 2005) and demonstrated PaCO_2_ levels (mmHg) of 42 ± 3 in the 40 6 h group, 68 ± 1 in the 65 6 h group, and 81 ± 4 in the 80 6 h group. All three groups were statistically different from each other (*p* < 0.05). Mean arterial pH values for the three groups were 7.42 ± 0.07 in the 40 6 h group and statistically lower in both the 65 6 h group, 7.30 ± 0.06, and the 80 6 h groups 7.25 ± 0.01, *p* < 0.05. pH levels were not different between the 65 6 h and the 80 6 h groups. All piglets had similar mean blood pressure and heart rate values (Fritz et al., 2005).

### Behavioral testing

The neurologic outcomes of neonates are a significant concern, given their increased risk of encountering learning deficits, attention issues, and intellectual impairment (Rogers and Hintz, 2016; Woythaler, 2019; Cortese et al., 2021). The specific contribution of HC to these deficits remains uncertain. While numerous behavioral and cognitive testing protocols have emerged in recent years for pigs (Gieling et al., 2011), most of them are not specifically designed for neonatal piglets and tend to emphasize repetitive tasks rather than assessing executive functioning. Therefore, we developed a novel testing system in piglets was developed to assess the higher cortical executive functions, involving task performance capacity, learning, and working memory. Gieling et al. (2011) recently reviewed modalities for behavioral testing in pigs and published criteria for evaluating these tests which included the following characteristics: The test must involve healthy animals; be detailed, stress-free, ecologically relevant, and standardized; allow for investigation of developmental effects; and be repeatable, automated, and complex but sensitive enough to detect subtle differences in cognitive abilities. Our test meets all of these criteria except for being automated. Our testing involved observing and recording a range of piglet movements and behavior so it was not automatable.

Piglets were tested daily starting the day after exposure to HC or NC by using a novel system to train and test newborn piglets in drinking milk from color-coded boxes (Extended Data Fig. 1-2). Piglets have dichromatic color vision (Chandler et al., 1999; Hendrickson and Hicks, 2002) allowing them to perceive blue, yellow, and white hues. They also possess a strong sense of smell (Gieling et al., 2011; Rorvang et al., 2023). To confirm that piglets were relying on their vision rather than their sense of smell to locate the milk-filled box, initial tests involved three sealed white boxes, with only one containing milk. The tight seal on the white boxes likely reduced the milk scent, making it challenging for the piglets to distinguish. However, when colored sealed boxes were introduced, the piglets successfully identified the milk-containing box after training.

Recovery NC and HC piglets were tested individually with one piglet in the pen at a time at zeitgeber time 1 or 1 h after the lights came on in the animal facility. Other piglets were temporarily moved from the communal pen. A feeding board was placed into their pen in the same location in the early morning before daily feedings were given. The feeding board consisted of a plank with three sealed, color-coded plastic boxes (blue, white, and yellow) attached to it, only one of which had milk in it. Before steps 1 and 4, each piglet underwent training by uncovering the milk-filled box once and allowing them to drink for 5 s. Following this single training phase, the piglet was positioned at the entrance of the pen and released. The piglet's objective was to find the box containing milk, uncover it, and consume the milk. The corresponding behaviors were evaluated using a standardized checklist with scores ranging from −2 to +3. The time to complete the task was also measured. To advance to the next step, a piglet had to successfully complete the previous one within 300 s. If a piglet did not achieve this goal within the time limit, the trial was halted, and the time was documented as 300 s. If the piglet failed to find and open the milk-filled box and drink within 300 s, they were shown the open milk-filled box again (i.e., were “trained” again) and retested. To evaluate the piglets’ capacity to learn, adapt, and remember (i.e., which color box contained the milk rather than relying on the position within the feeding system), we modified the testing conditions once the previous step had been accomplished successfully. These steps spanned the entire 7 d testing period and are outlined below and in Extended Data Figure 1-2.

Step 1

1. Milk was poured inside the blue box.
2. *Training phase*
3. Piglets were then tested twice daily.

Step 2

1. The position of the blue box on the feeding system was changed.
2. Piglets were no longer shown the open blue box with milk prior to testing.
3. Piglets were then tested twice daily.

Step 3

1. The position of the blue box was shifted again to the third position on the feeding board.
2. Piglets were no longer shown the open blue box with milk prior to testing.
3. Piglets were then tested twice daily.

Step 4

1. Milk was poured into the white container instead of the blue container.
2. *Training phase*
3. Piglets were then tested twice daily.

Scoring system:

+2 = opens the milk box and drinks
+1 = touches the milk box, and does not open it
+1 = does not approach other boxes
  0 = smells either of the other two boxes
−1 = tries to open either of the other two boxes
−2 = not interested in boxes at all

The maximum achievable score was +3, with the piglet opening the filled box, drinking, and not approaching or sniffing the other two boxes.

Behavioral scores per day for the two sessions were averaged and grouped as HC and NC results. The time to feed was also recorded for both sessions per day and grouped as HC and NC results. The two groups were compared via *t* tests.

### EEG protocol

Bipolar double-distance EEGs were performed on sedated piglets by placing 11 scalp electrodes after cleaning the skin with Nuprep and attaching the electrodes with Elefix. EEGs were continuously recorded digitally with a speed of 30 mm/s and an amplitude of 50 μV/mm. Electrode pairs of FP1-T3, FP2-T4, C3-O1, C4-O2, T3-O1, and T4-O2 were recorded with a reference pair of A1–A2 and a ground on the piglet's forehead. Each piglet served as their control. EEGs were performed on five piglets and continuously recorded before, during, and after exposure to 3 h of HC and at 7 d after exposure to HC (recovery piglets). Recordings were taken during the initial NC period, HC onset, HC full (15 min of added CO_2_ was considered “full HC”), and 1 h post HC and at 7 d of recovery. These piglets were sedated with 0.8 cc intramuscular dose (20 mg/kg/dose) of Nembutal (sodium 50 mg/1 cc solution) and monitored as the above recovery experimental protocol outlines. EEGs were also conducted on ventilated piglets exposed to 1 h of severe HC using the same aforementioned protocol. Recordings were made at three specific time points: during the NC phase onset, at the onset of HC, and at the peak of HC, which occurred 15 min into the HC period.

### Tissue preparation

#### Mitochondrial cell membrane

The cerebral cortices were homogenized and placed in an ice-cold isolation medium for the preparation of mitochondria using the methods of Booth and Clark (1978) and Lasso Pirot et al. (2007). Approximately 1 g of cortical tissue was homogenized in a Dounce-type glass homogenizer (total clearance, 0.1 mm) with 30 ml of fresh isolation medium. The homogenate was centrifuged for 3 min at 1,500 × *g*, and the supernatant was centrifuged for 10 min at 15,000 × *g* to provide the crude mitochondrial fraction and to separate any nuclei from the preparation. To purify mitochondria, the pellet was homogenized and placed on a Ficoll gradient, also to ensure that the final suspension was free of nuclei. The gradient was centrifuged for 30 min at 100,000 × *g*. The mitochondria pellet was washed and resuspended in the isolation medium. The purity of the mitochondrial fraction was documented by the absence of a specific cytosolic marker (S-100 β chain) and the presence of a specific mitochondrial marker, cytochrome *c* oxidase subunit IV in this fraction.

#### Synaptosomes

Synaptosomal purification from cortical neurons was performed by using a well-established method described by Booth and Clark (1978), Razdan et al. (1993), and Buonocore et al. (1999). In brief, cortical tissue was homogenized in an isolation medium (0.32 M sucrose/l mM potassium EDTA/lOmM Tris-HCl, pH7.4), followed by centrifugation to isolate a mitochondrial/synaptosomal pellet. Subsequently, this pellet was carefully loaded onto a discontinuous Ficoll/sucrose gradient and centrifuged again. Synaptosomes congregated at an interface between two gradient layers, while other cellular components were distributed throughout the gradient. After collection, the synaptosomes underwent a thorough washing process to eliminate any residual contaminants. Notably, synaptosomes obtained through this method exhibited high metabolic activity and minimal contamination (<4% nonsynaptosomal material, myelin, and “free” brain mitochondria) as confirmed by electron microscopy and enzyme assays (Booth and Clark, 1978). The synaptosomes were suspended in Krebs buffer medium (10 mM Tris-HEPES, 125 nM NaCl, 5 mM Kell, 1 mM CaCl_2_, 0.1 mM MgCl_2_, and 6 mM glucose, pH 7.4). Protein content was quantified following the Lowry method (Lowry et al., 1951).

#### Neuronal nuclei

Cerebral cortical neuronal nuclei were isolated and purified using a modification of the method of Giufrida et al. (1975). The nuclear fraction was purified by centrifugation at 53,000 × *g* for 60 min. The criteria used to define different types of nuclei are those described by Austoker et al. (1972) and assessed using a phase contrast microscope (Olympus). The neuronal nuclei were characterized by the presence of a centrally located nucleolus (one nucleolus/nucleus) compared with the presence of multiple nucleoli in the astrocytic and oligodendrocytic nuclei. The final nuclear preparation was devoid of any microsomal, mitochondrial, or plasma membrane contaminant, with purity for neuronal nuclei of ≥90% (Austoker et al., 1972).

#### Nuclear proteins

Nuclear proteins were separated from neuronal nuclei on a 12% SDS–PAGE gel and transferred to nitrocellulose paper (Ravishankar et al., 2001) for Western blotting techniques. Protein content was determined by the method of Lowry et al. (1951).

#### Cytosolic fractions

Cortical tissue was homogenized in 15 volumes of a medium containing 0.32 M sucrose, 10 mM Tris-HCl (pH 6.8), and 3 mM MgCl_2_. The homogenate was centrifuged at 850 × *g* for 10 min. The supernatant was centrifuged at 100,000 × *g* for 60 min to obtain the cytosolic fraction. All procedures were carried out at 0–4°C (Khurana et al., 2002).

#### Mitochondrial proteins

Mitochondrial proteins were prepared from mitochondrial membranes using the method of Lasso Pirot et al. (2007), for the determination of apoptotic protein density.

### Biochemical analysis

#### Lipid peroxidation products (fluorescent compounds and conjugated dienes)

Lipid peroxidation is a chain reaction in which reactive species interact with the polyunsaturated fatty acids in membrane lipids. This reaction results in the formation of intermediary products that continue to damage biological materials and finally results in chloroform-extractable lipophilic end products that contain innate fluorescent pigments (Ayala et al., 2014). These fluorescent compounds (FC) have not been fully chemically defined but are widely used as markers of lipid oxidation (Ivica and Wilhelm, 2014). In addition, when polyunsaturated fatty acids are oxidized, rearrangements of double bonds lead to the formation of membrane-conjugated dienes (CD) that are also a marker of lipid peroxidation (Kodali et al., 2020). To determine the production of FCs and CDs, we conducted measurements on synaptosomal membranes, which were extracted using 2:1 chloroform:methanol containing 0.005% β-hydroxytoluene and 0.5 mM EDTA according to the method of Folch et al. (1957). FC were measured using the method of Dillard and Tappel (1984). The fluorescence of the brain lipid extract was measured spectrofluorimetrically with an excitation wavelength of 360 nm and an emission wavelength of 435 nm. Sample intensity was compared with a quinine standard. CDs were determined as described by Recknagel and Glende (1984), in synaptosomal membrane fractions. Both FC and CD results were compared with the total brain lipid content and expressed per gram brain (lipid equivalent).

#### ATP and PCr

Cortical cerebral tissue concentrations of ATP and PCr were determined spectrophotometrically using the coupled enzyme assay by Lamprecht et al. (1973).

#### IP_3_ receptor binding

Inositol triphosphate (IP_3_) receptor binding was performed on neuronal nuclei in a medium containing 50 mM HEPES (pH 8.0), 2 mM EDTA, ^3^H- IP_3_ (7.5–100 nM), and 100 μg of nuclear protein. Nonspecific binding was determined in the presence of 10 μM unlabeled IP_3_. The IP_3_ receptor characteristics binding maximum (Bmax), the number of receptor sites and dissociation constant (Kd) were determined (Mishra et al., 2003).

#### High-affinity Ca^2+^-ATPase activity

The activity of Mg^2+^-dependent high-affinity calcium (Ca^2+^)-ATPase in nuclear membranes was determined using a modification of the methods of Ghandi and Ross (Mishra and Delivoria-Papadopoulos, 2001).

#### Intranuclear ^45^Ca^2+^ influx

The intranuclear ^45^Ca^2+^ influx was determined at 37°C for 120 s in a medium containing neuronal nucleus (150 μg protein), 1 μM ^45^Ca^2+^, and 1 mM ATP (Mishra and Delivoria-Papadopoulos, 2002).

#### CaM kinase IV activity

CaM kinase IV was determined according to Park and Soderling (1995) in neuronal nuclei.

#### Phosphorylated CREB Ser^133^

Expression of phosphorylated cAMP response element binding protein (pCREB) density was determined through previously described Western blotting techniques (Mishra and Delivoria-Papadopoulos, 2002; Fritz et al., 2005) on neuronal nuclear membranes. The membranes were incubated with anti-pCREB Ser^133^ antibodies. Specific Immunoreactivity was then detected by incubation with horseradish peroxidase–conjugated secondary antibody (Rockland Immunochemicals). Specific complexes were detected by enhanced chemiluminescence method using the ECL detection system (Amersham Pharmacia Biotech) and analyzed by imaging densitometry (GS-700 densitometer, Bio-Rad). The densitometric scanning data were expressed as autoradiographic values per immunoblot protein. Concomitant actin protein gels were run to demonstrate the consistent amounts of protein in each sample. Samples were run in duplicates. Proteins were expressed as absorbance/optical density (OD × mm^2^).

#### Apoptotic protein expression

Bax, Bcl-2, Bad, and Bcl-xl protein expression was determined on nuclear proteins separated from neuronal nuclei by SDS–polyacrylamide gel electrophoresis and transferred to nitrocellulose membranes using a wet *trans*-blotting system (Ravishankar et al., 2001), as well as on cytosolic (Halat, 1992) and mitochondrial proteins (Lasso Pirot et al., 2007). The membranes were incubated with polyclonal anti-Bax, anti-Bcl2, anti-Bad, or anti–Bcl-xl antibodies (Santa Cruz Biotechnology). Immunoreactivity was then detected by incubation with horseradish peroxidase–conjugated secondary antibody (Rockland Immunochemicals). Specific complexes were detected by enhanced chemiluminescence method using the ECL detection system (Amersham Pharmacia Biotech) and analyzed by imaging densitometry (GS-700 densitometer, Bio-Rad). The densitometric scanning data were expressed as autoradiographic values per immunoblot protein. Concomitant actin protein gels were run to demonstrate the consistent amounts of protein in each sample. Samples were run in duplicates. Proteins were expressed as absorbance/OD (OD × mm^2^).

#### Caspase-9 and caspase-3 activity

The activity of caspase-9 and caspase-3 was determined in cytosolic extracts using specific substrates according to Walker et al. (1994) and Khurana et al. (2002).

#### Caspase-3 and caspase-9 expression

Expression of caspase-9 and caspase-3 was also determined by the Western blotting techniques detailed above (Ravishankar et al., 2001; Khurana et al., 2002; Mishra et al., 2002), by using caspase-9 and caspase-3–specific antibodies (Santa Cruz Biotechnology).

#### Fragmentation of nuclear DNA

Deoxyribonucleic acid (DNA) fragmentation was determined from neuronal nuclei protein and separated into 1% agarose gel as described by Akhter et al. (2001).

### Statistical analysis

Data are presented as mean ± standard error of the mean (SEM) unless otherwise noted. Statistical analysis was performed using one-way analysis of variance (ANOVA) for multiple groups and two-tailed *t* tests for two groups by GraphPad Prism 9.5.1. For blood gas values, the pH, PaO_2_, and PaCO_2_ were plotted versus time, and a transformation was done by SigmaPlot software to determine the average blood gas value for that piglet experimental period. A *p*-value of <0.05 was considered statistically significant.

## Results

### Neonatal HC induces persistent abnormal neuronal cortical activity and behavioral deficits

**Figure 1.**
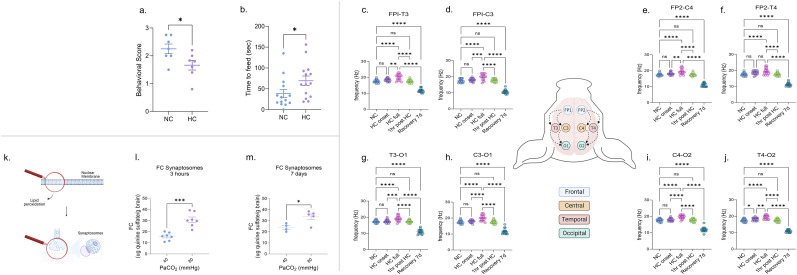
Neonatal HC induces persistent abnormal neuronal cortical activity and behavioral deficits. ***a***, Severe (PaCO_2_ 80 mmHg) HC piglets exhibited markedly inferior task scores and (***b***) experienced a prolonged time to complete the task compared with their NC counterparts. ***c–j***, Bipolar-distance EEGs showed that a 3 h exposure to severe HC significantly increased EEG frequencies in all channels, which normalized after 1 h of spontaneous recovery to room air. After a 7 d recovery period, the cortical activity globally decreased in the HC piglets compared with the baseline in all channels. ***k***, Depiction of peroxidation of cellular and subcellular neuronal membranes. ***l***, Measurement of indirect lipid peroxidation products from synaptosomes showed an increase in FC after 3 h of severe HC, ***m***, which remained elevated even 7 d after the initial exposure. The above noninstrumented chamber piglets were exposed to 3 h of severe (CO_2_, 80 mmHg) HC with and without 7 d of NC recovery after HC. Each group was compared with a group of sham piglets with a similar timeline of events except for the EEG studies where each HC piglet served as their control by using their initial NC period as the control. Extended Data Figure 1-1 demonstrates the experimental animal protocols for the piglets. Extended Data Figure 1-2 shows the representation of the milk-drinking training system for evaluating behavioral and cognitive function. Extended Data Figure 1-3 demonstrates the effect of 1 h of severe HC (PaCO_2_ 80 mmHg) on bipolar-distance EEGs. HC, hypercapnia; EEG, electroencephalogram; FC, fluorescent compounds; PaCO_2_, partial pressure of carbon dioxide; NC, normocapnia. Statistical analysis was performed using one-way ANOVA for multiple groups and two-tailed *t* tests for two groups by Prism statistical software, and the graph displays the mean ± SEM values; *p < 0.05, **p < 0.01, ***p < 0.001, ****p < 0.0001; *n* = 5–7/group. Part of this figure was created with BioRender.

Based on a previous study, which demonstrated that HC induced cortical injury in newborn piglets (Fritz et al., 2005), we hypothesized that exposure to HC would compromise cerebral cortex–associated cognitive functions. To test this hypothesis, newborn piglets were exposed to 3 h of severe HC (PaCO_2_ 80 mmHg) in a chamber and allowed to spontaneously recover before undergoing behavioral testing for 7 d (Extended Data Fig. 1-1) by using a novel paradigm of training newborn piglets to drink milk from color-coded boxes (Extended Data Fig. 1-2). Severe HC piglets demonstrated significantly worse task scores (Fig. 1*a*) and prolonged duration to task completion (Fig. 1*b*) than those of their NC counterparts. These data are consistent with an impairment in cortical executive function encompassing difficulties in decision-making, task switching, working memory, and visual recognition (Hofmann et al., 2012; Diamond, 2013), which persists for at least 7 d following the acute exposure to HC.

10.1523/ENEURO.0268-23.2023.f1-1 Extended Data Fig 1-1 The study included two main groups of newborn piglets: **(a)** Spontaneous recovery group: Non-instrumented piglets were exposed to three hours of severe HC (PaCO_2_ 80 mmHg) in a chamber, followed by seven days of recovery in room air. **(b)** Rapid resuscitation group: Ventilated, instrumented piglets were exposed to moderate (PaCO_2_ 65 mmHg) or severe (PaCO_2_ 80 mmHg) HC for one hour, followed by rapid resuscitation and an hour of NC ventilation. Each group was compared to a group of sham piglets with similar instrumentation and timeline of events, HC: Hypercapnia; PaCO_2_: Partial pressure of carbon dioxide; NC: Normocapnia. Figure created with BioRender. Download Extended Data Fig 1-1, TIF file.

10.1523/ENEURO.0268-23.2023.f1-2 Extended Data Fig 1-2 Schematic representation of the milk-drinking training system for evaluating cognitive function in newborn piglets using color-coded boxes. Non-instrumented piglets were exposed to three hours of severe HC (PaCO_2_ 80 mmHg) in a chamber, followed by seven days of recovery in room air. On the day after HC, piglets were trained and then tested twice daily on their ability to find, open and drink from a milk-filled box for seven days. Time to achieve that task was recorded. Piglets moved on to the next step once the prior task was achieved within 300 seconds. Each group was compared to a group of sham piglets with similar instrumentation and timeline of events HC: Hypercapnia; NC: Normocapnia. Figure created with BioRender. Download Extended Data Fig 1-2, TIF file.

10.1523/ENEURO.0268-23.2023.f1-3 Extended Data Fig 1-3 **(a-h)** Effect of a short duration (one hour) of severe HC (PaCO_2_ 80 mmHg) on bipolar-distance EEGs showed an increase in EEG frequencies in selected channels during one hour of HC. These piglets were exposed to severe (PaCO_2_: 80 mmHg) HC for one hour. Each piglet served as their own control with their initial NC period considered baseline. HC: Hypercapnia; PaCO_2_: Partial pressure of carbon dioxide; EEG: Electroencephalogram; NC: Normocapnia. Statistical analysis was performed using one-way analysis of variance for multiple groups by Prism statistical software, and the graph displays mean ± SEM values; * p<0.05, ** p<0.01, *** p<0.001; n=3-5/group. Download Extended Data Fig 1-3, TIF file.

**Figure 2.**
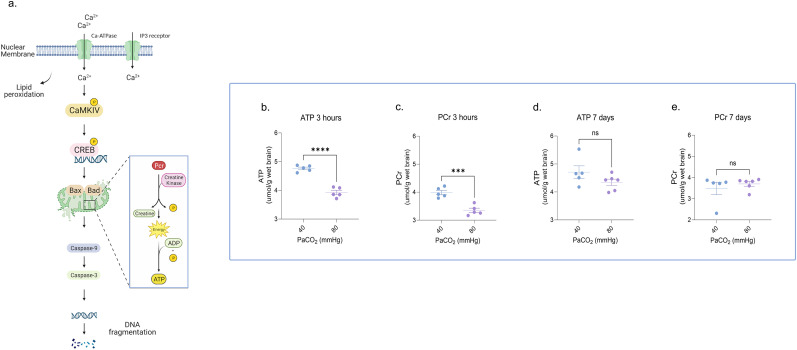
Neonatal HC–induced neuronal energy failure is transient. ***a***, Schematic representation illustrating the impact of peroxidation of neuronal membranes on energy demand. ***b,c***, Severe HC for 3 h transiently decreased tissue ATP and PCr levels in cortical neurons. ***d,e***, However, ATP and PCr levels in cortical neurons returned to normal after 7 d of recovery following the HC insult. The above noninstrumented chamber piglets were exposed to 3 h of severe (PaCO_2_ 80 mmHg) HC with and without 7 d of NC recovery post HC. Each group was compared with a group of NC sham piglets with a similar timeline of events. HC, hypercapnia; ATP, adenosine triphosphate; PCr, phosphocreatine; PaCO_2_, partial pressure of carbon dioxide; NC, normocapnia. Statistical analysis was performed using one-way ANOVA for multiple groups and two-tailed *t* tests for two groups by Prism statistical software, and the graph displays the mean ± SEM values; *p < 0.05, **p < 0.01, ***p < 0.001, ****p < 0.0001; *n* = 5–6/group. Part of this figure was created with BioRender.

**Figure 3.**
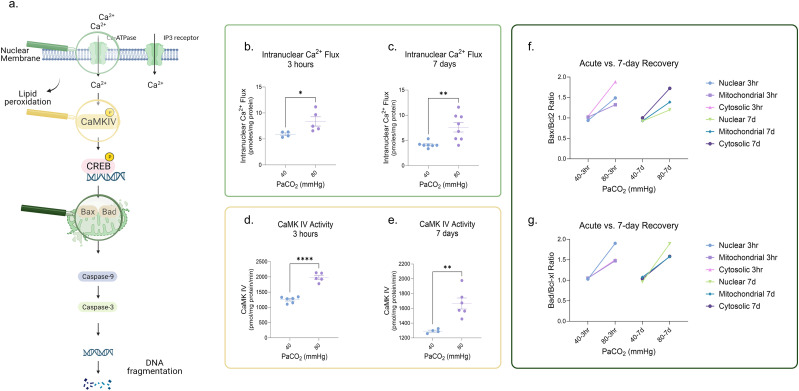
Neonatal HC induces persistent dysregulation of calcium-dependent proapoptotic signaling in the cerebral cortex. ***a***, Schematic representation of the HC-induced activation of Ca^2+^-dependent pathways and its consequential impact on the expression of apoptotic proteins. HC increased both (***b,c***) intranuclear Ca^2+^ influx and (***d,e***) CaMK IV activity after 3 h of exposure which persisted 7 d after the insult. ***f,g***, Three hours of HC also increased ratios of Bax/Bcl-2 and of Bad/Bcl-xl in the nucleus, cytosol, and mitochondria, which persisted at 7 d of recovery after HC, especially in the cytosol (Bax) and nucleus (Bad). The above noninstrumented chamber piglets were exposed to 3 h of severe (PaCO_2_ 80 mmHg) HC with and without 7 d of NC recovery post HC. Each group was compared with a group of NC sham piglets with a similar timeline of events. Extended Data Figure 3-1 illustrates the impact of HC on nuclear Ca^2+^ signaling and Extended Data Figure 3-2 demonstrates the expression of pro- and antiapoptotic proteins following HC exposure. Representative Western blots of Bax, Bad, Bcl-2, and Bcl-xl expression are shown in Extended Data Figure 3-3. Ca^2+^, calcium; HC, hypercapnia; CaMK IV, calmodulin-dependent protein kinase IV; NC, normocapnia, PaCO_2_, partial pressure of carbon dioxide. Statistical analysis was performed using one-way ANOVA for multiple groups and two-tailed *t* tests for two groups by Prism statistical software, and the graph displays the mean ± SEM values; *p < 0.05, **p < 0.01, ***p < 0.001, ****p < 0.0001; *n* = 5–7/group. Part of this figure was created with BioRender.

We then sought to determine whether HC induces a persistent dysfunction in cortical neurons by measuring bipolar double-distance EEGs before, during, and after HC. We found that 3 h of severe HC significantly increased EEG frequencies (Fig. 1*c–j*), with a clear effect in all channels; 1 h of severe HC produced a similar increase, although in fewer channels (Fig. 1-3). While the 3 h of HC-induced EEG changes normalized after 1 h of spontaneous recovery in room air, after the 7 d recovery period, the same piglets demonstrated that cortical electrical activity had decreased globally in all EEG channels, as compared with baseline. This is indicative of persistent neuronal dysfunction following HC.

### Neonatal HC induces lipid peroxidation in cortical synaptosomes

Lipid peroxidation is a key phenomenon linking oxidative stress to the integrity of membrane-bound receptors, altering neuronal membrane activity, and contributing to neuronal cell death (Mishra et al., 1990, 2000; Halliwell, 1999; Fig. 1*k*). We quantified lipid peroxidation end products FC in cortical synaptosomes as potential indicators of oxidative stress and cellular damage within synaptic structures suggesting an impaired synaptic function of the cerebral cortex. Three hours of HC resulted in the production of FCs (Fig. 1*l*) that remained elevated 7 d later (Fig. 1*m*). In summary, our data suggest that HC-induced mechanisms elicit a lasting oxidative injury that could impair synaptic signaling and potentially affect gross neuronal activity and behavior.

### Neonatal HC–induced neuronal energy failure is transient

As HC can induce acidosis, which may retard oxidative phosphorylation (Cady et al., 1987), we hypothesized that HC may decrease cortical tissue levels of ATP and PCr (Fig. 2*a*). Furthermore, given the persistence of lipid peroxidation induced by HC, we also posited that neurons may also demonstrate a persistent increase in energy demand. We found significantly decreased levels of ATP and PCr following 3 h of severe HC (Fig. 2*b,c*). However, the HC-induced acute energy failure was restored to baseline 7 d later (Fig. 2*d,e*), suggesting normalization of neuronal bioenergetics despite the persistence of lipid peroxidation.

### Neonatal HC induces persistent dysregulation of calcium-dependent proapoptotic signaling cerebral cortex

We next sought to investigate the downstream molecular effects of HC that may contribute to persistent neuronal dysfunction. HC induces cellular acidosis linked to intracellular and intranuclear Ca^2+^ influx (Malinovskii and Kostesha, 1993; Gerasimenko et al., 1996; Mishra et al., 2003). In a previous study, it was reported that acute exposure to HC induced activation of calcium-dependent pathways, with upregulation of calcium/calmodulin-dependent kinase IV (CaMK IV) activity, CREB phosphorylation, and increased expression of proapoptotic protein Bax (Fritz et al., 2005; Fritz and Delivoria-Papadopoulos, 2006; Fig. 3*a*). Therefore, we sought to determine whether dysregulated Ca^2+^-dependent signaling also played a role in the persistence of the HC-induced neuronal defects. While we did not find a consistent effect of HC on the activity of the nuclear high-affinity Ca^2+^-ATPase enzyme (Extended Data Fig. 3-1a), we did find that HC increased the binding capacity (Bmax) and affinity (1/Kd) of the IP_3_ receptor in the nuclear membrane, which was accompanied by an increase in intranuclear Ca^2+^ influx (Extended Data Fig. 3-1b–d). Like the persistent lipid peroxidation following HC, HC also increased both intranuclear Ca^2+^ flux (Fig. 3*b,c*) and tyrosine-phosphorylated CaMK IV activity (Fig. 3*d,e*) acutely and chronically.

10.1523/ENEURO.0268-23.2023.f3-1 Extended Data Fig 3-1 These figures illustrate the impact of HC on nuclear Ca^2+^ signaling. **(a)** The activity of the nuclear high-affinity Ca^2+^-ATPase enzyme did not change during severe HC. However, HC led to a significant increase in the **(b)** Bmax and **(c)** affinity (1/Kd) of the IP_3_ receptor in the nuclear membrane. **(d)** This enhanced IP_3_ receptor function is accompanied by an increase in intranuclear Ca^2+^ influx. Each of the above groups of piglets were exposed to either moderate (PaCO_2_ 65 mmHg) or severe (PaCO_2_ 80 mmHg) HC for six hours. Each group was compared to a group of NC sham piglets with similar instrumentation and timeline of events. HC: Hypercapnia; Ca^2+^: Calcium; Bmax: Binding maximum; Kd: disassociation constant; IP_3_: inositol triphosphate; PaCO_2_: Partial pressure of carbon dioxide; NC: Normocapnia. Statistical analysis was performed using one-way analysis of variance for multiple groups and two-tailed t-tests for two groups by Prism statistical software, and the graph displays mean ± SEM values; * p<0.05, ** p<0.01, *** p<0.001, **** p<0.0001; n=4-6/group. Download Extended Data Fig 3-1, TIF file.

10.1523/ENEURO.0268-23.2023.f3-2 Extended Data Fig 3-2 **(a-l)** Expression of proapoptotic (Bax and Bad) and antiapoptotic (Bcl-2 and Bcl-xl) proteins in distinct neuronal compartments following HC exposure. Expression profiles were measured in neuronal nuclei, cytosol, and mitochondria and demonstrated a significant increase in Bax and Bad in all the cellular compartments during severe HC which persisted for seven days except for nuclear Bax concentrations. These non-instrumented chamber piglets were exposed to three hours of severe (PaCO_2_: 80 mmHg) HC with and without seven days of NC recovery after HC. Each group was compared to a group of sham NC piglets with a similar instrumentation and timeline of events. HC: Hypercapnia; PaCO_2_: Partial pressure of carbon dioxide; NC: Normocapnia. Statistical analysis was performed using one-way analysis of variance for multiple groups by Prism statistical software, and the graph displays mean ± SEM values; * p<0.05, ** p<0.01, *** p<0.001, **** p<0.0001; n=3-9/group. Download Extended Data Fig 3-2, TIF file.

10.1523/ENEURO.0268-23.2023.f3-3 Extended Data Fig 3-3 Shows representative western blots of Bax, Bad, Bcl-2, and Bcl-xl in neuronal nuclei, cytosol, and mitochondria following HC exposure. Download Extended Data Fig 3-3, TIF file.

Increased nuclear Ca^2+^ activates CaM kinases, including CaMK IV resulting in apoptotic neuronal death (Delivoria-Papadopoulos and Mishra, 2004). Based on our observation of the HC-induced increases in nuclear Ca^2+^ influx, CaMK IV activity, and expression of phosphorylated CREB, we aimed to define the downstream effect of HC on apoptotic protein expression and determine the cellular compartments most sensitive to these changes. We measured the expression of proapoptotic (Bax and Bad) and antiapoptotic (Bcl-2 and Bcl-xl) proteins in neuronal nuclei, cytosol, and mitochondria (Extended Data Fig. 3-2a–l). There is a known correlation between an increasing ratio of proapoptotic/antiapoptotic proteins shifting the cellular balance toward apoptotic death (Raisova et al., 2001). Three hours of HC increased ratios of Bax/Bcl-2 and of Bad/Bcl-xl in the nucleus, cytosol, and mitochondria, which persisted at 7 d of recovery after HC (Fig. 3*f,g*), with Bax/Bcl-2 ratios in the cytoplasm and Bad/Bcl-xl ratios in the nucleus being the most sensitive to the effects of HC at 7 d of recovery. These data suggest that HC may predispose cortical neurons to undergo apoptotic cell death well after acute HC exposure.

### Neonatal HC causes neuronal apoptosis and DNA fragmentation

**Figure 4.**
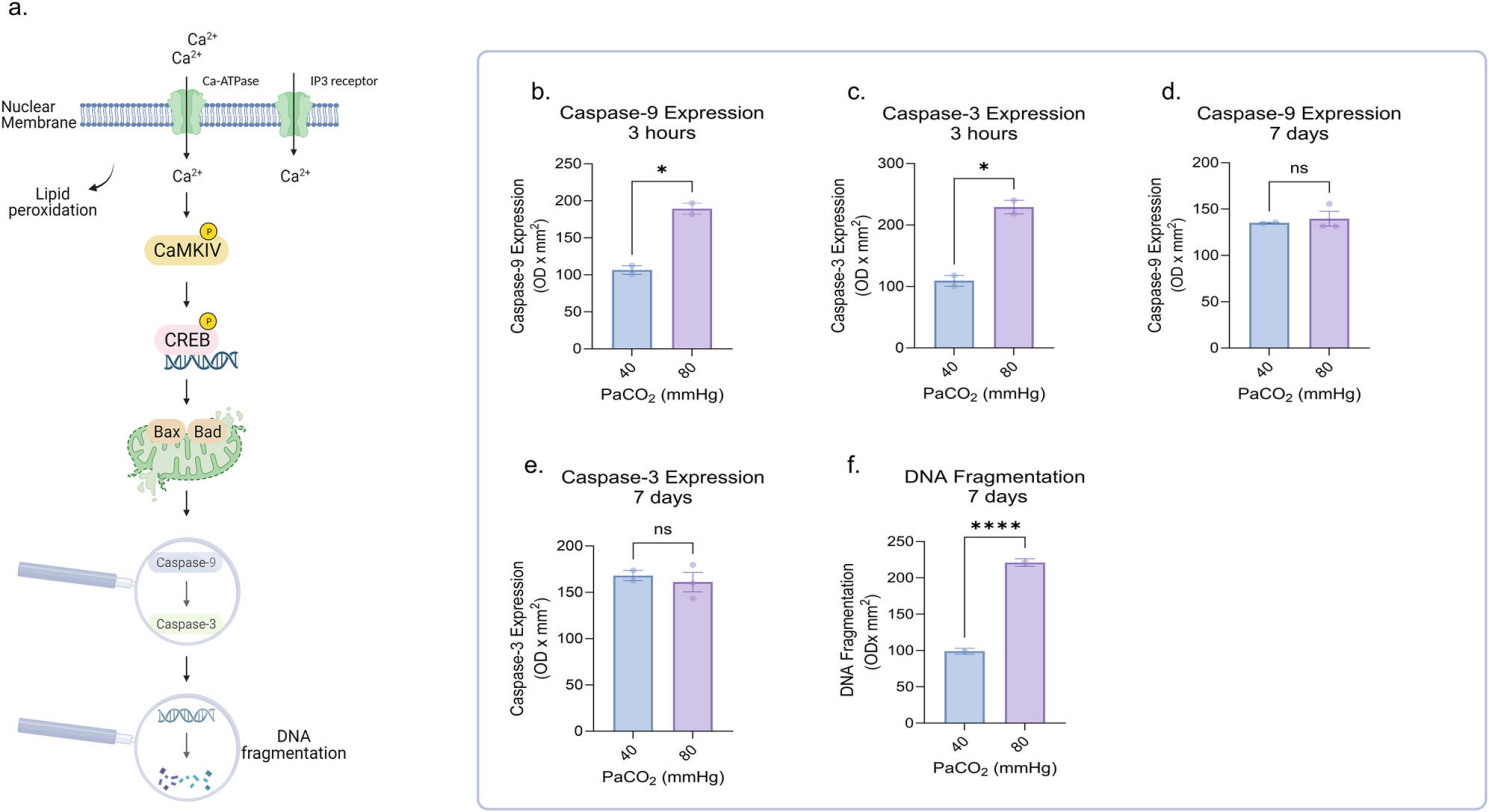
Neonatal HC causes neuronal apoptosis and DNA fragmentation. ***a***, The schematic representation illustrates the cell death process where activation of proapoptotic signals leads to the activation of caspases, which, in turn, results in nuclear DNA fragmentation and prolonged cell death. ***b–e***, Three hours of HC increased levels of caspase-9 and caspase-3 expression, which returned to baseline after 7 d of recovery under NC conditions; (***f***) however, DNA fragmentation was elevated at 7 d after HC compared with NC controls. The above noninstrumented chamber piglets were exposed to 3 h of severe (PaCO_2_ 80 mmHg) HC with and without 7 d of NC recovery post HC. Each group was compared with a group of NC sham piglets with a similar timeline of events. Extended Data Figure 4-1 illustrates the effect of HC on caspase-9 and caspase-3 expression and activity and on nuclear DNA fragmentation. Representative Western blots of caspase-9 and caspase-3 expression are shown in Extended Data Figures 4-3 and 4-4 and of DNA fragmentation in Extended Data Figure 4-2. DNA, deoxyribonucleic acid; HC, hypercapnia; NC, normocapnia; PaCO_2_, partial pressure of carbon dioxide. Statistical analysis was performed using one-way ANOVA for multiple groups and two-tailed *t* tests for two groups by Prism statistical software, and the graph displays the mean ± SEM values; *p < 0.05, **p < 0.01, ***p < 0.001, ****p < 0.0001; *n* = 5/group. Part of this figure was created with BioRender.

The activation of proapoptotic signals promotes cell death by activating caspases that lead to nuclear DNA fragmentation and programmed cell death (Ravishankar et al., 2001; Fig. 4*a*). Therefore, we quantified the expression of caspase 9 and 3 in cortical neurons in addition to nuclear DNA fragmentation. HC was capable of acutely increasing caspase-9 and caspase-3 expression levels, as well as nuclear DNA fragmentation (Fig. 4*b,c*; Extended Data Fig. 4-1a–f). At 7 d following HC, caspase-9 and caspase-3 expression levels normalized (Fig. 4*d,e*); however, DNA fragmentation remained elevated (Fig. 4*f*). Of note, there was no increase in caspase-9 or caspase-3 activity after 3 h of HC or at 7 d recovery after HC (Extended Data Fig. 4-1g,h). Importantly, rather than a “ladder pattern” of fragmentation, all piglets exposed to HC displayed a smear pattern of small molecular weight DNA fragments between 100 and 12,000 bp.

10.1523/ENEURO.0268-23.2023.f4-1 Extended Data Fig 4-1 The effect of HC on of caspase-9 and caspase-3 expression levels and activity and on nuclear DNA fragmentation in cortical neurons showed **(a,b,d,e)** an increase in caspase-9 and -3 expression following both six hours and one hour of HC, **(g-h)** but no significant changes in caspase-9 or caspase-3 activity were observed after three hours of HC or following the seven day recovery period. **(c,f)** There was a significant increase in DNA Fragmentation after one and six hours of HC. Each of the above groups of piglets was exposed to either moderate (PaCO_2_: 65mmHg) or severe (PaCO_2_: 80mmHg) HC for one, three or six hours. Each group was compared to a group of NC sham piglets with similar instrumentation and timeline of events. DNA: Deoxyribonucleic acid; HC: Hypercapnia; PaCO_2_: Partial pressure of carbon dioxide; NC: Normocapnia. Statistical analysis was performed using one-way analysis of variance for multiple groups by Prism statistical software, and the graph displays mean ± SEM values; * p<0.05, ** p<0.01, *** p<0.001, **** p<0.0001; n=5-6/group. Download Extended Data Fig 4-1, TIF file.

10.1523/ENEURO.0268-23.2023.f4-2 Extended Data Fig 4-2 Shows representative western blots of DNA Fragmentation in 6 hour and 1 hour HC and NC piglets. Download Extended Data Fig 4-2, TIF file.

10.1523/ENEURO.0268-23.2023.f4-3 Extended Data Fig 4-3 Demonstrates representative western blots of caspase-9 and caspase-3 expression in 6 hour and 1 hour HC piglets and NC piglets. Download Extended Data Fig 4-3, TIF file.

10.1523/ENEURO.0268-23.2023.f4-4 Extended Data Fig 4-4 Shows representative western blots of caspase-9 and caspase-3 expression in recovery HC and NC piglets. Download Extended Data Fig 4-4, TIF file.

### Rapid resuscitation to normocapnia does not prevent HC-induced cortical injury

**Figure 5.**
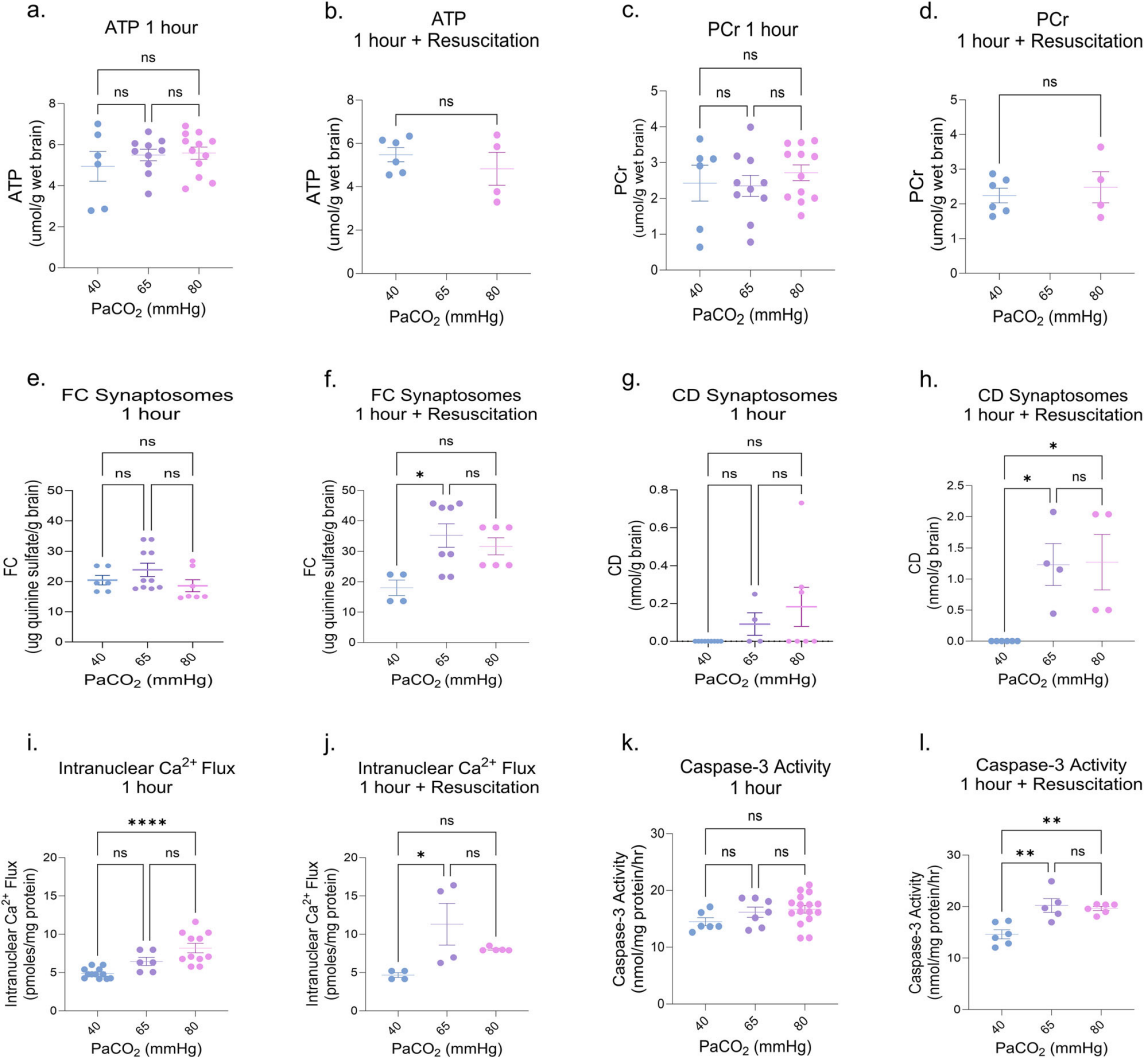
Rapid resuscitation to normocapnia does not prevent HC-induced cortical injury. Rapid normalization of PaCO_2_ following 1 h of HC in newborn piglets (***a–d***) showed no effect on ATP and PCr levels either after 1 h of HC or following the rapid resuscitation of HC. However, following rapid resuscitation after HC, there was a delayed elevation of (***e–h***) synaptosomal lipid peroxidation products, FC and CDs, (***i–l***) intranuclear Ca^2+^ influx, and caspase-3 activity. Each of the above groups of ventilated piglets was exposed to either 1 h of moderate (PaCO_2_, 65 mmHg) or severe (PaCO_2_, 80 mmHg) HC or 1 h of HC followed by 1 h of rapid resuscitation. Each group was compared with a group of NC sham piglets with similar instrumentation and timeline of events. PaCO_2_, partial pressure of carbon dioxide; HC, hypercapnia; ATP, adenosine triphosphate; PCr, phosphocreatine; FC, fluorescent compounds; CD, conjugated dienes; Ca^2+^, calcium; NC, normocapnia. Statistical analysis was performed using one-way ANOVA for multiple groups and two-tailed *t* tests for two groups by Prism statistical software, and the graph displays the mean ± SEM values; *p < 0.05, **p < 0.01, ***p < 0.001, ****p < 0.0001; *n* = 4–10/group.

Given that human newborns frequently undergo resuscitation when their PaCO_2_ levels rise acutely, our study aimed to explore the potential preventive effects of rapidly correcting PaCO_2_ levels (“resuscitation”) on HC-induced injury. We designed an experimental paradigm that involved mechanical ventilation, exposing newborn piglets to 1 h of HC, followed by rapid adjustment of their PaCO_2_ to normal levels (Extended Data Fig. 1-1b). Interestingly, this protocol did not affect cellular energetics either at 1 h of HC or following the rapid normalization of HC (Fig. 5*a–d*). However, a delayed onset of synaptosomal membrane lipid peroxidation was observed as measured by the production FC and CDs after 1 h of NC resuscitation, but not during 1 h of HC (Fig. 5*e–h*), further suggesting a dissociation between energy failure and the mechanisms responsible for synaptosomal lipid peroxidation following HC. Similarly, despite a rapid normalization of PaCO_2_, a delayed increase in Ca^2+^ levels and caspase-3 activity were noted, suggesting that cellular dysregulation is likely to persist even after a shorter “dose” of HC and resuscitation (Fig. 5*i–l*). These findings prompt further inquiry into the therapeutic approach for HC, specifically examining the comparative merits of gradual versus rapid PaCO_2_ adjustment.

In the present study, we determined the functional and molecular responses of cortical neurons in newborn piglets to different degrees and lengths of HC as well as to the effects of 7 d of spontaneous NC, normoxic recovery after HC, and rapid resuscitation after HC.

## Discussion

Sick newborns often experience HC while undergoing positive pressure ventilation during the neonatal period. The use of our large animal model allows for addressing the effects of HC in a manner that cannot be rivaled by most clinical studies, as hospitalized patients often bear multiple risk factors for poor neurodevelopmental outcomes that are challenging to control (e.g., prematurity, hypoxia, infections, hypotension, congenital anomalies, and chromosomal abnormalities). For instance, in a recent landmark multicenter trial (Permissive Hypercapnia in Extremely Low Birth Weight Infants), no differences in neurodevelopmental outcomes were detected between patients randomized to “mild” (i.e., PaCO_2_ 40–60) versus “high” (i.e., PaCO_2_ 55–75) target PaCO_2_ groups (Thome et al., 2017). While this study was not powered to detect these differences, further post hoc analysis reinforced that the degree of HC in these neonates was likely more associated with disease severity than a true independent variable (Thome et al., 2018). Our use of a large animal model of normoxic HC allows us to specifically investigate the effects of isolated HC on the brain acutely with and without rapid resuscitation and 7 d following injury.

Building upon previous studies by Fritz et al. (2005) and Fritz and Delivoria-Papadopoulos (2006), we found that a relatively short duration of HC (3 h) was capable of impairing learning and cognitive functioning as well as EEG alterations for 7 d after exposure. The results are also consistent with studies in a newborn rat pup model that found long-term behavioral and neuronal deficits following a combined hypoxic and hypercapnic neonatal insult (Tachibana et al., 2013). Our study's unique focus on the effects of HC, specifically, more accurately reflects common NICU practice in which patients are often afforded normoxia with isolated HC. Furthermore, we also show here that HC induces persistent dysregulation in neuronal calcium-dependent signaling pathways that ultimately culminate in proapoptotic signals, providing a plausible mechanism by which permissive HC, alone, may produce lasting neuronal injury.

Increased intranuclear Ca^2+^ may be one of the main drivers and perpetuating mechanisms for neuronal cell injury after HC. The kinetics of changes in Ca^2+^ flow and activation of second messengers appear to be dissociated from HC-induced energy failure, suggesting an alternative mechanism linking HC to dysregulated calcium signaling. During HC, there is a decrease in extracellular pH and intracellular pH (Cady et al., 1987), which results in an increase in free cytosolic Ca^2+^ (Siesjo et al., 1993). HC-induced cortical membrane lipid peroxidation from acidosis or from increased cerebral blood flow and oxygenation may alter neuronal membrane Ca^2+^ influx. In addition, HC-induced increased intracellular hydrogen ion concentrations may compete with free cytosolic Ca^2+^ (Siesjo et al., 1993), which may in turn lead to an increase in intranuclear Ca^2+^ influx.

We also demonstrate here an HC-induced increase activity of the IP_3_ receptor of the nuclear membrane, with activation CaMK IV, which is predominantly located in the nucleus and activates transcription factors such as CREB by phosphorylating its Ser^133^ site (pCREB), a necessary step in CREB-mediated transcription (Sun et al., 1994). Activated pCREB binds to the DNA regulatory sequence cAMP response element with CREB binding protein and p300 (Chrivia et al., 1993). Interestingly, we found the persistence of increased CaMK IV 7 d after recovery. Further downstream, we found that the pro- and antiapoptotic ratio of Bax/Bcl-2 and Bad/Bcl-xl remained elevated as well. While HC appears to trigger caspase activity that may be elicited by activation of the CaMK IV pathway, the activation, as expected, does not persist a week after the exposure to HC. Furthermore, DNA fragmentation lacks the typical “ladder type” of fragmentation commonly seen in apoptosis, suggesting that ongoing random DNA breakage (e.g., free radical damage and necrosis) is also present in these cells following HC.

Our studies also have given insight into the effects of resuscitation on HC-induced brain injury. Since our piglets were normoxic and normotensive, we were able to isolate the effects of resuscitation after HC alone. One hour of NC resuscitation after HC resulted in the normalization of some cortical biochemical parameters, but the persistence of many and an increase or initiation of other parameters—increased production of lipid peroxidation products, a partial increase in intranuclear Ca^2+^ influx, and increased caspase-3 activity—indicated that the process of neuronal injury initiated by 1 h of HC was ongoing. Although some of the biochemical parameters improved after rapid NC resuscitation, there may be other modes of resuscitation that may be more beneficial to ameliorate the HC-induced brain injury seen in the newborn piglet, such as a slower lowering of CO_2_ or the addition of buffers to improve intracellular and extracellular pH.

Given these results and evidence of both harm and benefit in previous studies, we consider permissive HC in neonates as less than an ideal choice and far from a panacea in the management of ill neonates. The net benefits of avoiding ventilator-associated lung injury and barotrauma when trialing permissive HC, especially when reaching impasse situations such as maximum ventilatory settings, should be weighed against the short and long-term risks that it poses, including but not limited to cortical neuronal injury, intraventricular hemorrhage, hypoxic–ischemic encephalopathy, and necrotizing enterocolitis (Thome et al., 2015; Wong et al., 2022). The present study provides new insights into the complex effects of HC on neuronal processes, with significant ramifications for ongoing neurodevelopment. Every effort should be made to avoid the deleterious effect of HC, even permissive, unless the counter choice is devastating in very sick neonates in which ventilatory strategies are extremely challenging. In those cases, clinicians should target PaCO_2_ levels consistent with data from prior randomized controlled trials (Mariani et al., 1999; Carlo et al., 2002; SUPPORT Study Group of the Eunice Kennedy Shriver NICHD Neonatal Research Network et al., 2010; Dunn et al., 2011) that have shown some benefit while avoiding sudden changes or extremes of PaCO_2_ (Fabres et al., 2007).

### Study limitations

We acknowledge several limitations to our study. While our investigation primarily focused on the cerebral cortex, it is important to recognize that HC may have an impact on various other brain areas as well. Similarly, with the use of a large animal piglet model of HC, while providing superior experimental control and brain-similar anatomical features, we must be cautious in generalizing our data to the human newborn brain. While piglets are a valuable translational model due to their physiological and anatomical similarities to human infants, it is essential to acknowledge that the findings may not directly translate to other species or humans. The extrapolation of these results into other contexts should be done with caution. In addition, we used fresh and frozen brain tissues for the biochemical assays, which limited histological analysis requiring paraformaldehyde fixation; however, we were able to investigate nearly every step of the apoptotic pathway leading to DNA fragmentation. The extrapolation of these results into other contexts should be done with caution. The current study focused on the short-term effects of HC in newborn piglets up to 7 d after the insult. Long-term observations would be valuable to assess the lasting impact of hypercapnic conditions on neurodevelopment outcomes.

Despite these limitations, the use of piglets as a translational model in studying HC is of great significance, bridging the gap between preclinical and clinical research. Furthermore, future studies incorporating histological analyses and gene sequencing will contribute to a more comprehensive understanding of the implications of HC on neonatal neurodevelopment.

## Synthesis

Reviewing Editor: Benjamin Deneen, Baylor College of Medicine Decisions are customarily a result of the Reviewing Editor and the peer reviewers coming together and discussing their recommendations until a consensus is reached. When revisions are invited, a fact-based synthesis statement explaining their decision and outlining what is needed to prepare a revision will be listed below. The following reviewer(s) agreed to reveal their identity: Stephen A. Back, Tara DeSilva.

Overall the reviewers felt this paper of interest to the field and provides important new insights on neonatal hypercapnia on neonatal brain development using piglet models. While these findings are of interest, both reviewers feel revisions are in order for this manuscript to be published. Accordingly, it was felt that there was an over reliance on western blotting and that some histopathology should be included in the analysis to supplement these data. Also, greater details concerning the methods and clarification of how this study is distinct from prior piglet studies on this subject.

## Author Response

Point by point response to reviewers:

Reviewer 1 This study addresses an understudied and clinically significant problem related to the potential injurious roles of neonatal hypercapnia on neonatal brain development. The study has a number of strengths, particularly that it employs a gyrencephalic large pre-clinical animal model that has been previously studied in related contexts as a neonatal brain injury model.

Author Response: We appreciate the positive comments by Reviewer 1.

The study raises a number of questions:

1. What does this study add from this groups previous studies in piglets in 2005. The conclusion from the 2005 study was that hypercapnia is deleterious to the brain. It would be useful to summarize what additional innovative approaches are utilized here to strengthen the conclusions of the present study. It is important to discuss how the present study is simply not confirmatory since some similar questions are addressed (e.g. ATP and PCr levels) related to energy failure and cell death mechanisms. The present study does look at longer term outcomes for these and other measures and should be discussed in the context of prior studies.

Author Response: In the 2005 study, we examined the effects of hypercapnia on the cerebral cortex of newborn piglets, revealing that six hours of elevated CO2 resulted in energy failure, alterations in neuronal enzyme activity, and increased apoptotic protein expression, suggesting potential harm to the newborn brain. However, these biochemical findings were primarily obtained in the acute setting, very soon after the hypercapnic exposure. Our current study builds upon these data by focusing more on the downstream effects of a hypercapnic insult, with a goal of improving translatability. To this end, this paper's hypercapnia protocol was modified from the original in the following manner:

1. As a primary goal was to incorporate functional assessments of the cerebral cortex through EEG recordings and behavioral testing long after the initial insult, we needed to deliver a significant insult with low long-term mortality, and no prolonged instrumentation (to reduce the risk of infection or iatrogenesis).

2. With a focus on translatability of our findings, we concluded that three hours of hypercapnia will more closely mimic acute short-duration clinical scenarios in the neonatal ICU (e.g., acute deterioration in mechanically ventilated patients, pneumothorax, endotracheal tube dislodgement, post operative ventilatory management and other similar scenarios within the 3-hour range). This was adopted as our primary duration of intervention.

3. We also utilized one-hour exposures to hypercapnia with and without rapid CO2 correction to achieve normocapnia, mimicking acute respiratory failure scenarios in the delivery room and neonatal ICUs, to investigate the impact of rapid resuscitation.

4. In this paper we investigated the impact of hypercapnia on neuronal lipid peroxidation in the cerebral cortex which added more information on the pathogenesis of hypercapnia-induced cortical injury. In addition, we fully characterized step-by-step the apoptotic cascade following hypercapnia including the calcium signaling, the caspases and until the final step, the DNA fragmentation.

In summary, while our present study does build upon our 2005 study, the scope is larger and uses clinically relevant experimental paradigms to more closely approximate ICU scenarios. We have updated our Introduction and Methods to reflect this - see highlighted text in the revised manuscript.

2. What was the basis for the hypercapnia model? Why did the authors select a 3-hour exposure to severe hypercapnia? It is important to address whether this exposure reflects a clinically significant scenario. Alternatively, can the exposure be justified if piglets are relatively more resistant to hypercapnia than human infants.

Author Response: We utilized the three-hour experimental paradigm of hypercapnia because:

1. Three-hour duration of hypercapnia mimics acute clinical scenarios in the neonatal ICU (acute deterioration in mechanically ventilated patients, endotracheal tube dislodgement, post operative ventilatory adjustments and other similar scenarios within the 3-hour range). Relevant guidelines and practices can be found in this clinical manual: Goldsmith, Karotkin, Suresh, and Keszler, (2016). Assisted Ventilation of the Neonate: Evidence-Based Approach to Newborn Respiratory Care, 6th Edition.

2. Our study incorporated moderate and severe degrees of hypercapnia, to explore the possibility of considering moderate hypercapnia as permissive in the clinical setting.

3. To facilitate behavioral assessments seven days after the initial hypercapnic insult, we needed to ensure the survival of the piglets. Prolonged hypercapnic exposure would have required additional interventions, potentially increasing the risk of infection due to the introduction of extra instrumentation.

Finally, to our knowledge, the resistance of neonatal piglets to hypercapnia in comparison to human infants is unexplored.

3. The behavioral test is novel, but what is it measuring...memory, color recognition (do piglets have color vision?). More detail on the rationale for the test and the type of memory being tested is needed? Is this a hippocampally based learning task, or selective for other brain regions such as the olfactory or visual cortex? Is there any precedent in the literature for similar types of testing in piglets or other higher mammals? Author Response: We utilized a behavioral testing protocol which encompasses distinct cognitive domains, such as learning, visual recognition, olfactory response, pattern recognition, and problem solving, making it a valuable representation of cortical executive functioning. Specifically, we initially ensured that the piglets would be unable to perform the tasks based on their olfactory ability by performing the test using boxes of the same color. Please refer to the relevant section in methodology. Piglets do have dichromatic colored vision enabling them to recognize white, blue, and yellow hues [PMID: 12076087, 11397262] which were incorporated into our behavioral test protocol. Despite the numerous recent protocols on behavioral and cognitive testing in pigs, most of these protocols are not tailored for newborn piglets and tend to prioritize repetitive tasks over the evaluation of executive functioning. We included these clarification points in the revised manuscript.

4. Clarification of the behavioral testing protocol is needed. It seems that the piglets were tested twice daily (and results averaged) and the tests conducted over seven days. Figure 1a should provide a diagram illustrating the testing protocol...when was it initiated after HC, how many days of training trials before testing was begun and for how long? Author Response: We provide detailed description of the behavioral assessment and for improved clarity. We have also included a new visual diagram illustrating the testing protocol:

This new diagram appears as Figure 1-2 in the revised manuscript.

Specifically, in the behavioral testing protocol, the feeding board comprised three sealed, color-coded plastic boxes (blue, white, and yellow), with only one containing milk. Each piglet, one day following the HC exposure, underwent a training session until they accomplished the task, during which the milk-filled box was uncovered once, allowing them to drink for five seconds. After this initial training episode, the piglet was positioned at the entrance of the pen and released, with the task of locating the milk-containing box, removing the cover, and consuming it. The time taken to complete this task and the associated behaviors were recorded twice a day. To assess the piglets' ability to learn, adapt, and remember (i.e., identifying the color of the box containing milk rather than relying on its position within the feeding system), we modified the testing conditions once the previous step had been successfully accomplished. These steps were carried out over the entire seven-day testing period.

5. More detail is needed about the conjugated dienes and fluorescent compounds (FCs). Are these measured in cortical synaptosomal preparations? What is the significance of measuring FCs? Are they lipid peroxidation products or some other class of compounds derived from proteins or nucleic acids? Author Response: In evaluating lipid peroxidation within the cerebral cortex, we quantified its indirect products (fluorescent compounds, FCs and conjugated dienes CDs), in cortical synaptosomes. We opted to measure FCs and CDs in cortical synaptosomal preparations due to their potential to serve as markers of oxidative stress and cellular damage within synaptic structures, thus indicating potential impairment of synaptic function in the cerebral cortex.

Lipid peroxidation is a chain reaction involving reactive species and polyunsaturated fatty acids in membrane lipids, leading to the formation of intermediary products. These products can cause damage to biological materials and result in the creation of chloroform-extractable, lipophilic end products that contain innate fluorescent pigments (the FCs), widely used as markers of lipid oxidation. Additionally, the oxidation of polyunsaturated fatty acids leads to the formation of membrane conjugated dienes (CDs), also used as markers of lipid peroxidation.

In the updated version of our paper, we've integrated these explanatory details.

1. Supplemental Table 2: are there any significant differences among the groups? Author Response: Other than the hypercapnia and pH, there were no significant differences between the groups in the physiological data. To ensure that our experimental groups are comparable, we compared the physiological parameters of normocapnic and hypercapnic piglet groups (pH, blood pressure, heart rate, PaO2 and CO2). Our comparative analysis indicates that aside from differences in CO2 levels and pH, no other differences were observed between the groups. We have included the statistical comparisons in the Supplemental Table 2, now titled "Table 2".

2. In general, the text on the figures is very small. The figures need to be redone with attention to the journal guidelines for font sizes. The figures are not legible when they are enlarged in the PDF (especially figures 1, 2, 3 and 5). It is not possible to fully evaluate figures 1 and 2 due to the severe pixelation.

Author Response: Apologies for the poor resolution in the initial PDF submission. We have now uploaded the revised version of the final high-resolution figures and text according to the eNeuro guidelines, ensuring the highest possible resolution for improved legibility.

Reviewer 2 The investigators report some potentially interesting findings with important clinical impact to the field on vulnerability of the newborn infant and the potential untoward effects of standard procedures used for neonates. Overall the findings are interesting but the results sections does not present a synthesized story of how and why the specific experiments were performed. For example, it is not clear what type of behavior or cognition the different colored boxes for drinking milk is representing. Is this a cortical driven- behavior. All of the biochemistry is being performed in the cortex so it would be important to understand if the biochemistry of the neurons represents the behavior. In the same figure the investigators describe the milk boxes behavior and EEG, it is not clear how these behaviors relate to each other and if these neurons are represented in the biochemical analysis.

Author Response: We would like to thank Reviewer 2 for the overall positive comments and the thoughtful feedback. In response to this feedback, we have revised the manuscript, clarifying the rationale behind the experimental paradigms. As a result, we have created a more coherent narrative that clearly establishes the connections between the behavioral experiments, EEG recordings, and the biochemical analysis of the cerebral cortex.

Based on our previous work on the biochemical changes in the cortical neurons following hypercapnia:

1. We initially investigated whether the HC-induced cortical biochemical alterations were associated with behavioral deficits related to cognition, memory, and executive functions. To do this, we designed a battery of tests, revealing compromised cortical executive functioning due to HC.

2. Next, our objective was to determine whether HC had a direct and acute effect on the cerebral cortex or if it resulted from a delayed injury originating from other brain regions, possibly involving structures like basal ganglia or subcortical white matter through neurodegenerating mechanisms over a period of hours or days. Indeed, we demonstrated acute EEG alterations immediately following the onset of HC, indicating that elevated CO2 levels directly and acutely affected cortical neurons.

We acknowledge that executive functioning may involve circuitry beyond the cerebral cortex, such as basal ganglia and thalamus. To fully address the underlying mechanisms induced by hypercapnia, extensive studying of these neurocircuits is necessary. In this study, we primarily focus on the cerebral cortex as it is considered the primary center of executive functioning (PMID: 4408280, 32958176, 33640395, 35289723). Our data suggest that hypercapnia acutely and directly impacts cortical activity, with cortical electrographic changes appearing within minutes from the hypercapnia administration.

Based on the cortical neurobehavioral assessment and electrographic changes following hypercapnia, we focused our biochemical analysis on the neurons of the cerebral cortex. We demonstrated alignment of cortical processes through these assessments, suggesting that hypercapnia impacts the cerebral cortex.

Concerning "what the behavioral test findings are representing" we have now provided a more detailed explanation. To define the impact of hypercapnia on the cerebral cortical function, we assessed cortical-linked neurobehavior such as cognition, executive functioning, and memory. Specifically, we utilized an assessment battery to define:

1. Learning and memory (the piglet remembers the color of the food-container) 2. Task performance (the piglet will need to uncover the box to find the food) 3. Object and pattern recognition (the piglet remembers the color and the position of the food container) 4. Problem solving (the piglet identifies the changes in position and colors of the food container and understands that uncovering the container is required to access the food).

The battery of behavioral tests listed above assesses the executive functioning capacity of the piglets, a process linked to the cerebral cortex.

In the results section, the investigators state they are using a severe HC model. More elaboration would be necessary to understand why this model is being used and how it relates to the human condition. Perhaps this could be better explained in the introduction.

Author Response: Please see our response to Reviewer 1 in which we addressed this concern.

Furthermore, there are lack of details to support the data that is presented. Moreover, there are no data images to support the data bar graphs presented in all of the figures. It appears most of the data comes from western blots or agarose blots, but there are no representative images necessary to support the validity of the data.

Author Response: We have now added representative images of the blots as extended figure files.

Lastly, if the neurons are indeed stressed as the authors surmise, it would be beneficial to show some type of histopathological staining to support this premise.

Author Response: We acknowledge that a neuropathological analysis would significantly add value to this paper. However, for our experimental protocol we could only utilize fresh cortical tissue rapidly harvested within seconds particularly for the lipid peroxidation markers. In contrast, for a neuropathological analysis we would require perfusion of the piglets with formalin which would increase the number of experimental animals to a non-affordable cost based on our initial funding budget. However, we are now planning on including extensive and rigorous neuropathological assessment following hypercapnia in our next project.

1) In Figure 1, the figure legend states there are n=3-5 mice per group however, Figure 1b and c show 7 individual data points for each group.

Author Response: We updated the figure legend in Figure 1 to accurately reflect the number of piglets (n=7) per group as shown in Figure 1b and c.

2) In Fig. d-k the individual data points are extremely difficult to read.

Author Response: We have addressed the issue, and in the revised figures (d-k), we have improved the clarity and readability of individual data points for better visualization.

3) Characterization of the synaptosome purity would be necessary to properly evaluate the synaptosome data in Fig. 1m and n. Was the entire cortex taken for the prep excluding the hind brain and cerebellum. More clarification is needed in the methods section of the paper.

Author Response: Our group has consistently purified synaptosomes from cortical neurons using a well-established method outlined by Booth and Clark (PMID: 743245, 10537074, 8382100). With this method, the resulting synaptosomes were metabolically active and demonstrated minimal contamination, with less than 4% non-synaptic material, their as their metabolic activity and purity was validated through electron microscopy and enzyme assays.

In response to the concerns raised by Reviewer 2 regarding the extraction process, I can confirm that we took great care to ensure that only the cerebral cortex was prepared for analysis, with a thorough examination conducted to exclude any cerebellum or hindbrain tissue from the samples. This approach was taken after rapidly removing the cerebral cortices rapidly within seconds. The cortices were divided in half. The first half was used for cellular fraction isolation and the other half was promptly frozen in liquid nitrogen and stored at -80{degree sign}C for subsequent analysis of ATP, PCr, and lipid peroxidation products. The above reflects the revised version of the paper.

4) In Fig. 4 and the results section it states that caspase-9 and caspase-3 expression were measured. There are no details describing how this was measured. Representative images of what was measured should ie western blot is necessary.

Author Response: We added representative images obtained from Western blot analysis. To avoid overcrowding of the main figures, we included the images as extended figures. Furthermore, a dedicated section regarding the biochemical analysis of Caspases-9 and -3 expression has been added to the methods section.

5) Bax proteins were also measured by western blotting, but no representative images are shown.

Author Response: We have incorporated representative images derived from Western blot analysis. To avoid overcrowding of the main figures, we included the images as an extended figure.

6) The methods section states that DNA fragmentation was determined from neuronal nuclei and separated on 1% agarose gel, but no representative gels are shown.

Author Response: We have addressed this concern by incorporating representative agarose gel images illustrating DNA fragmentation. To avoid overcrowding of the main figures, we included the images as an extended figure.
