# Supplementary figures and images for: Hypercapnia Causes Injury of the Cerebral Cortex and Cognitive Deficits in Newborn Piglets
Source: eNeuro. 2024 Mar 1;11(3):ENEURO.0268-23.2023. doi: 10.1523/ENEURO.0268-23.2023 (PMC10913040; doi:10.1523/ENEURO.0268-23.2023)

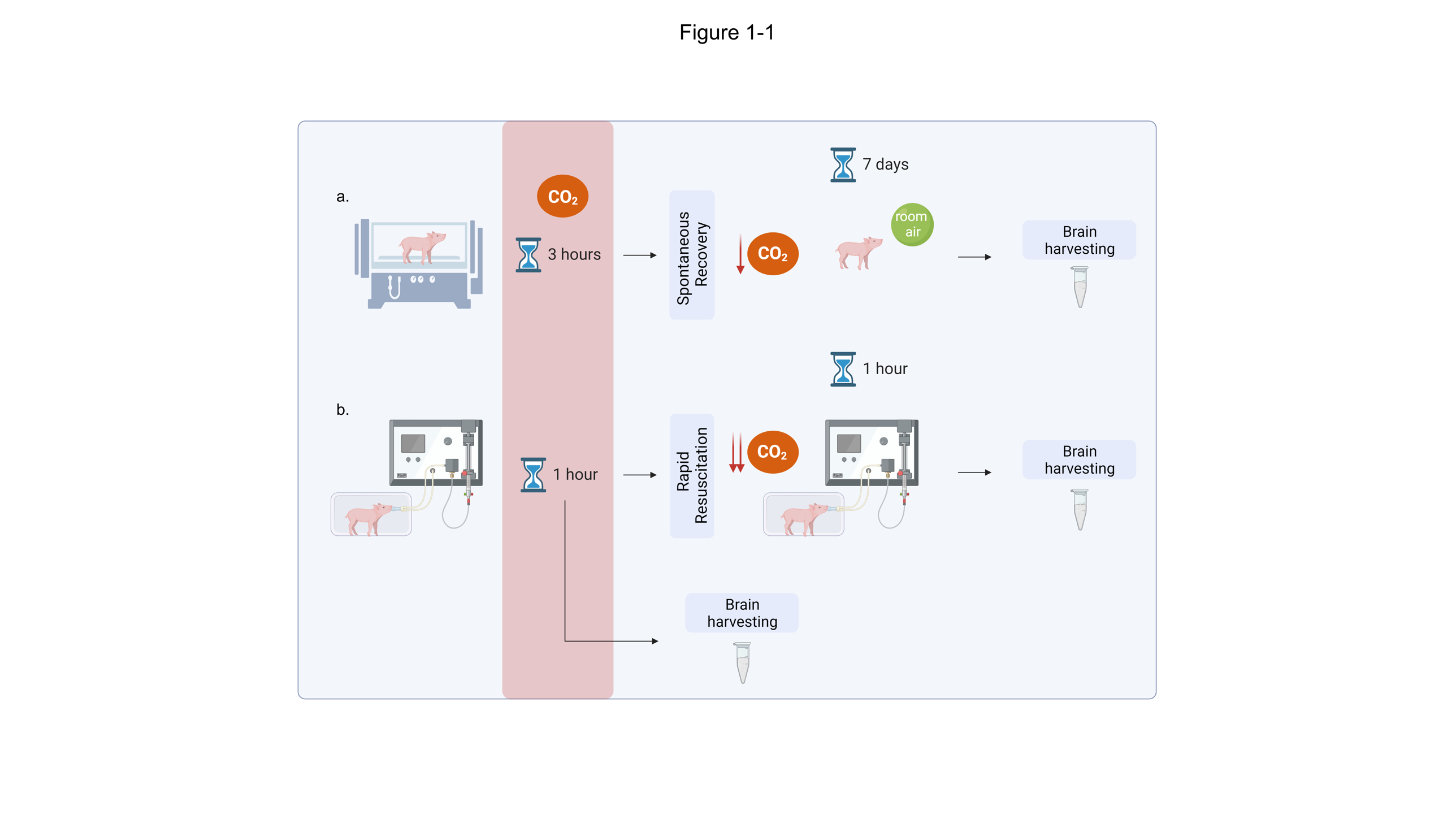

Supplement: Extended Data Fig 1-1 — The study included two main groups of newborn piglets: (a) Spontaneous recovery group: Non-instrumented piglets were exposed to three hours of severe HC (PaCO2 80 mmHg) in a chamber, followed by seven days of recovery in room air. (b) Rapid resuscitation group: Ventilated, instrumented piglets were exposed to moderate (PaCO2 65 mmHg) or severe (PaCO2 80 mmHg) HC for one hour, followed by rapid resuscitation and an hour of NC ventilation. Each group was compared to a group of sham piglets with similar instrumentation and timeline of events, HC: Hypercapnia; PaCO2: Partial pressure of carbon dioxide; NC: Normocapnia. Figure created with BioRender. Download Extended Data Fig 1-1, TIF file. [file eneuro-11-ENEURO.0268-23.2023-s002.tif]

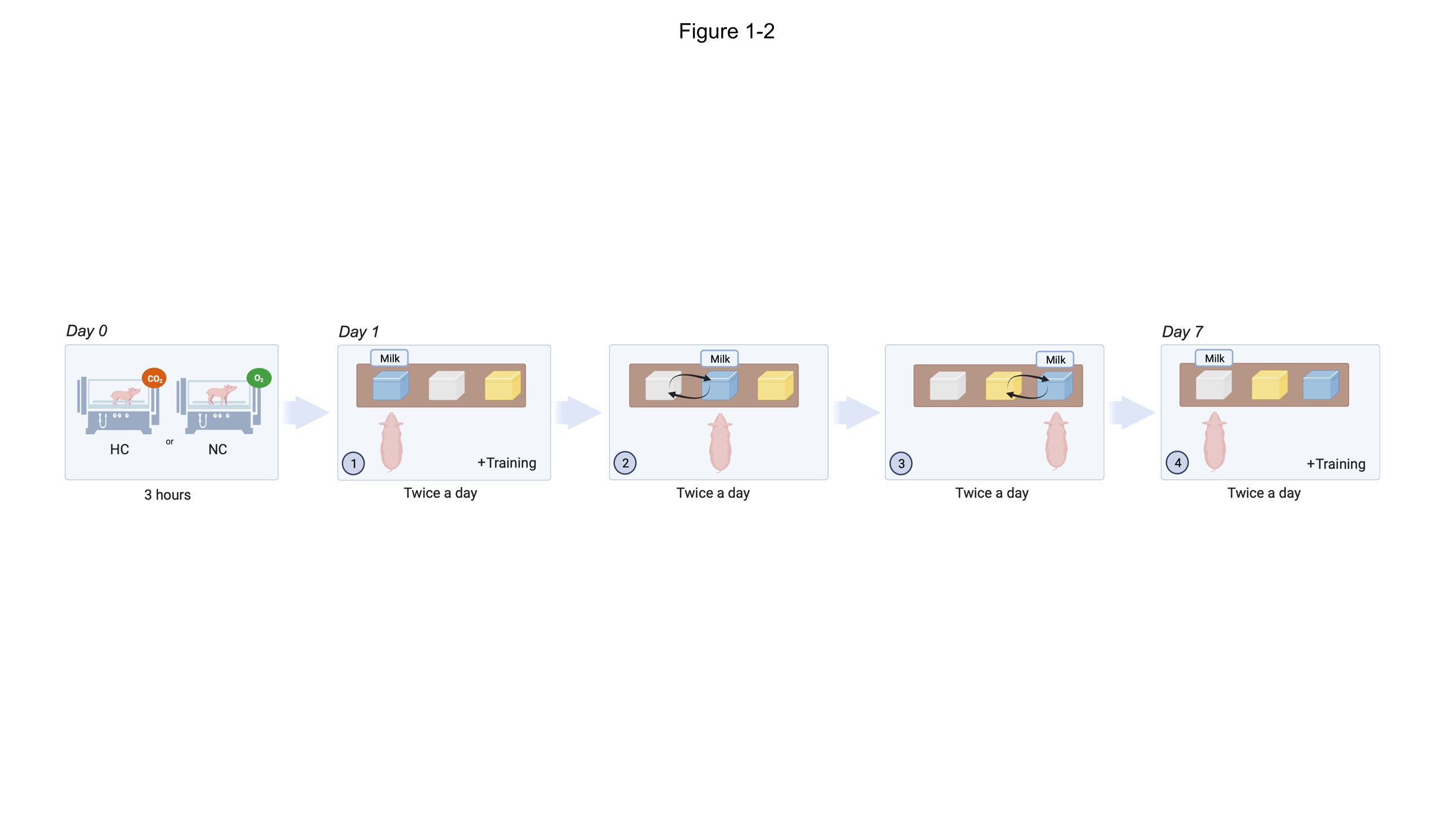

Supplement: Extended Data Fig 1-2 — Schematic representation of the milk-drinking training system for evaluating cognitive function in newborn piglets using color-coded boxes. Non-instrumented piglets were exposed to three hours of severe HC (PaCO2 80 mmHg) in a chamber, followed by seven days of recovery in room air. On the day after HC, piglets were trained and then tested twice daily on their ability to find, open and drink from a milk-filled box for seven days. Time to achieve that task was recorded. Piglets moved on to the next step once the prior task was achieved within 300 seconds. Each group was compared to a group of sham piglets with similar instrumentation and timeline of events HC: Hypercapnia; NC: Normocapnia. Figure created with BioRender. Download Extended Data Fig 1-2, TIF file. [file eneuro-11-ENEURO.0268-23.2023-s003.tif]

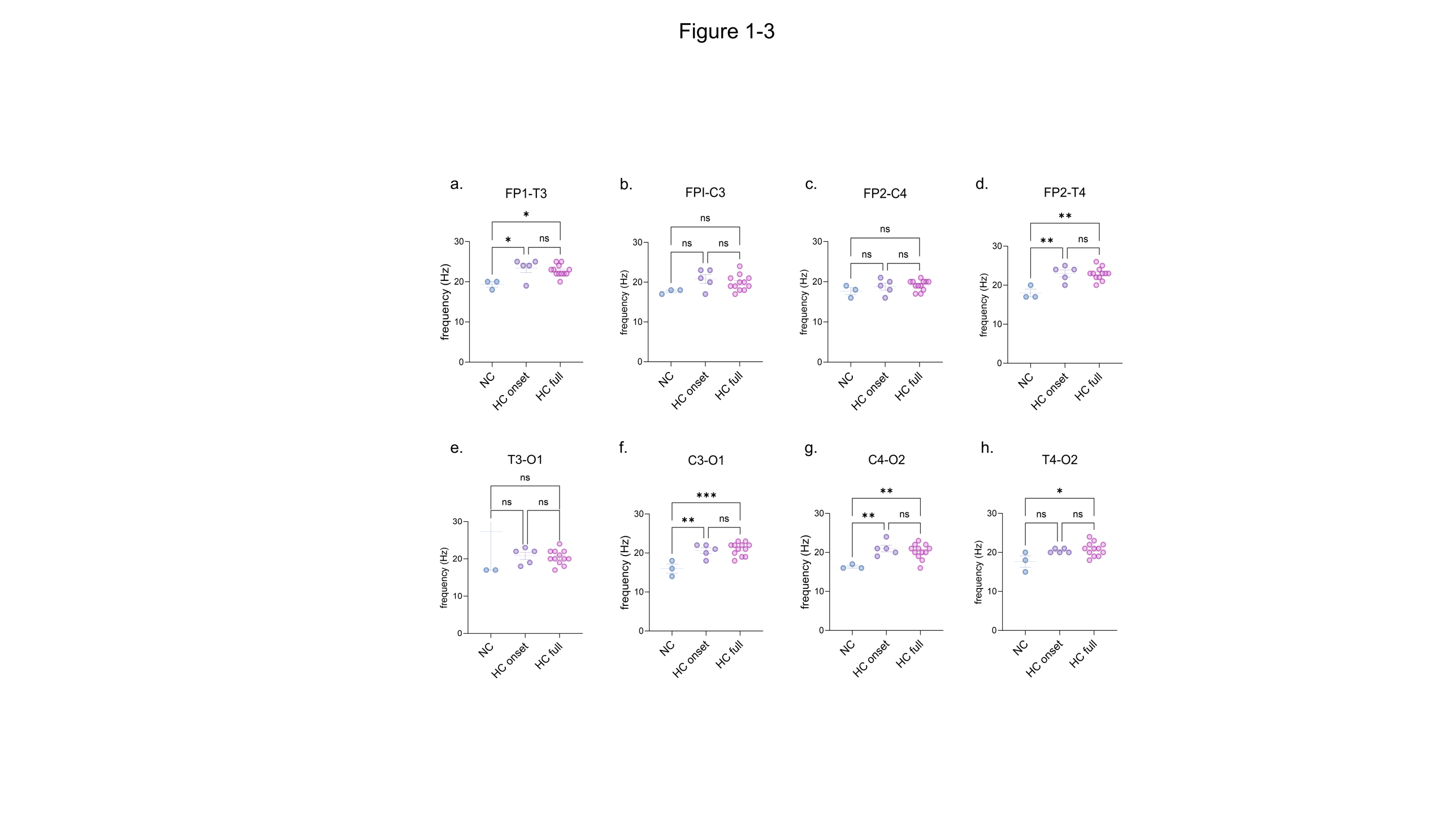

Supplement: Extended Data Fig 1-3 — (a-h) Effect of a short duration (one hour) of severe HC (PaCO2 80 mmHg) on bipolar-distance EEGs showed an increase in EEG frequencies in selected channels during one hour of HC. These piglets were exposed to severe (PaCO2: 80 mmHg) HC for one hour. Each piglet served as their own control with their initial NC period considered baseline. HC: Hypercapnia; PaCO2: Partial pressure of carbon dioxide; EEG: Electroencephalogram; NC: Normocapnia. Statistical analysis was performed using one-way analysis of variance for multiple groups by Prism statistical software, and the graph displays mean ± SEM values; * p<0.05, ** p<0.01, *** p<0.001; n=3-5/group. Download Extended Data Fig 1-3, TIF file. [file eneuro-11-ENEURO.0268-23.2023-s004.tif]

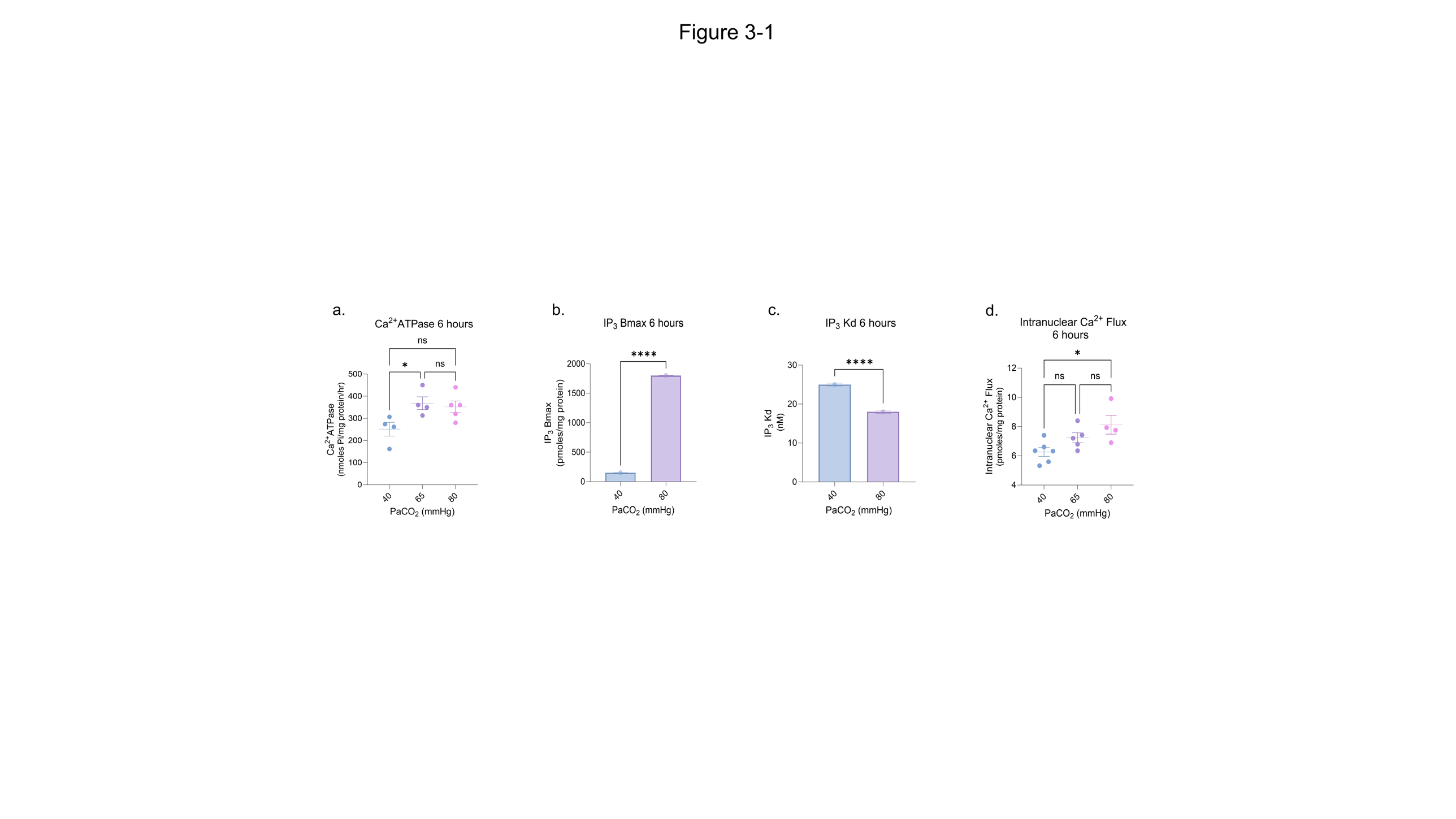

Supplement: Extended Data Fig 3-1 — These figures illustrate the impact of HC on nuclear Ca2+ signaling. (a) The activity of the nuclear high-affinity Ca2+-ATPase enzyme did not change during severe HC. However, HC led to a significant increase in the (b) Bmax and (c) affinity (1/Kd) of the IP3 receptor in the nuclear membrane. (d) This enhanced IP3 receptor function is accompanied by an increase in intranuclear Ca2+ influx. Each of the above groups of piglets were exposed to either moderate (PaCO2 65 mmHg) or severe (PaCO2 80 mmHg) HC for six hours. Each group was compared to a group of NC sham piglets with similar instrumentation and timeline of events. HC: Hypercapnia; Ca2+: Calcium; Bmax: Binding maximum; Kd: disassociation constant; IP3: inositol triphosphate; PaCO2: Partial pressure of carbon dioxide; NC: Normocapnia. Statistical analysis was performed using one-way analysis of variance for multiple groups and two-tailed t-tests for two groups by Prism statistical software, and the graph displays mean ± SEM values; * p<0.05, ** p<0.01, *** p<0.001, **** p<0.0001; n=4-6/group. Download Extended Data Fig 3-1, TIF file. [file eneuro-11-ENEURO.0268-23.2023-s005.tif]

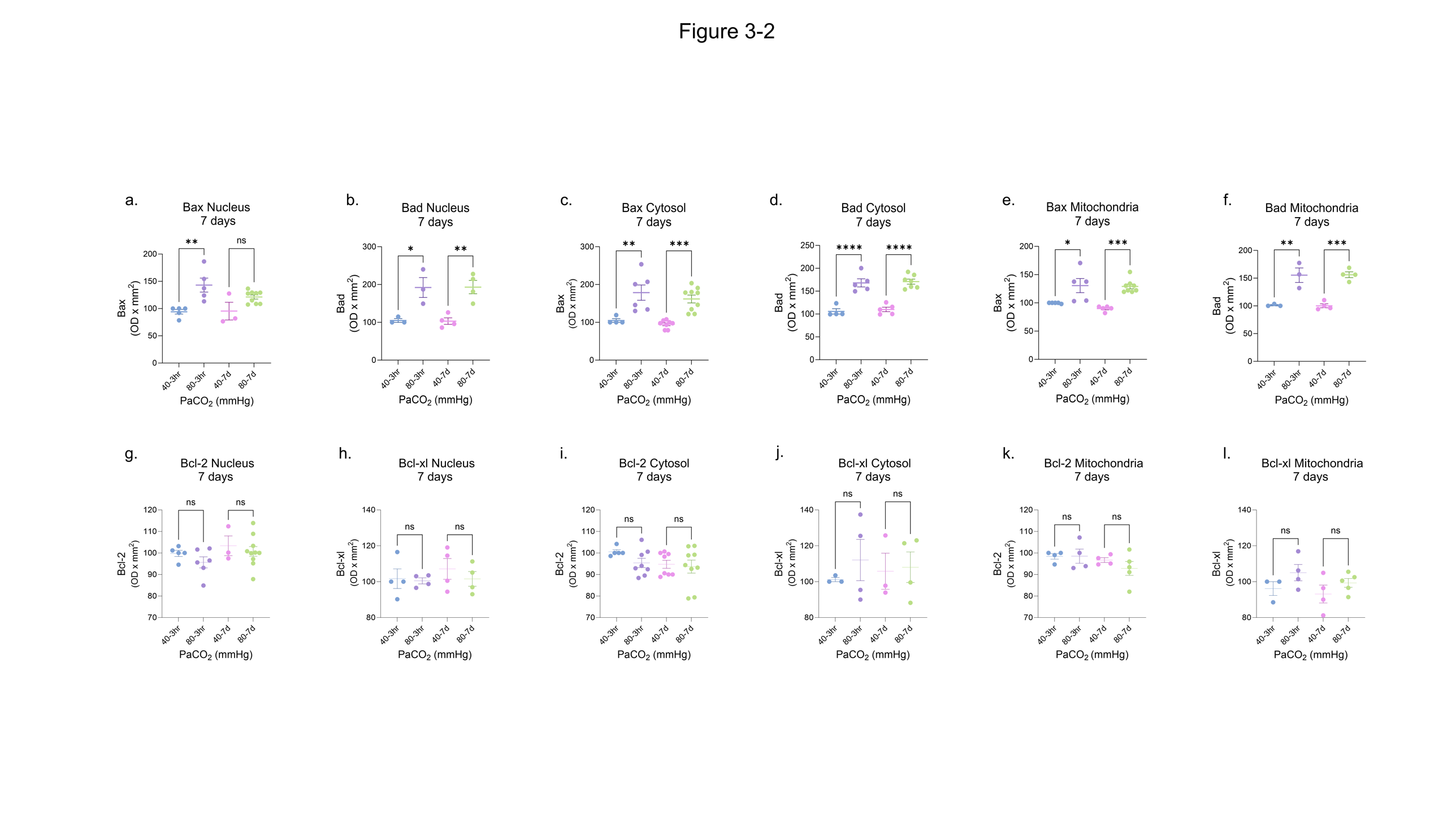

Supplement: Extended Data Fig 3-2 — (a-l) Expression of proapoptotic (Bax and Bad) and antiapoptotic (Bcl-2 and Bcl-xl) proteins in distinct neuronal compartments following HC exposure. Expression profiles were measured in neuronal nuclei, cytosol, and mitochondria and demonstrated a significant increase in Bax and Bad in all the cellular compartments during severe HC which persisted for seven days except for nuclear Bax concentrations. These non-instrumented chamber piglets were exposed to three hours of severe (PaCO2: 80 mmHg) HC with and without seven days of NC recovery after HC. Each group was compared to a group of sham NC piglets with a similar instrumentation and timeline of events. HC: Hypercapnia; PaCO2: Partial pressure of carbon dioxide; NC: Normocapnia. Statistical analysis was performed using one-way analysis of variance for multiple groups by Prism statistical software, and the graph displays mean ± SEM values; * p<0.05, ** p<0.01, *** p<0.001, **** p<0.0001; n=3-9/group. Download Extended Data Fig 3-2, TIF file. [file eneuro-11-ENEURO.0268-23.2023-s006.tif]

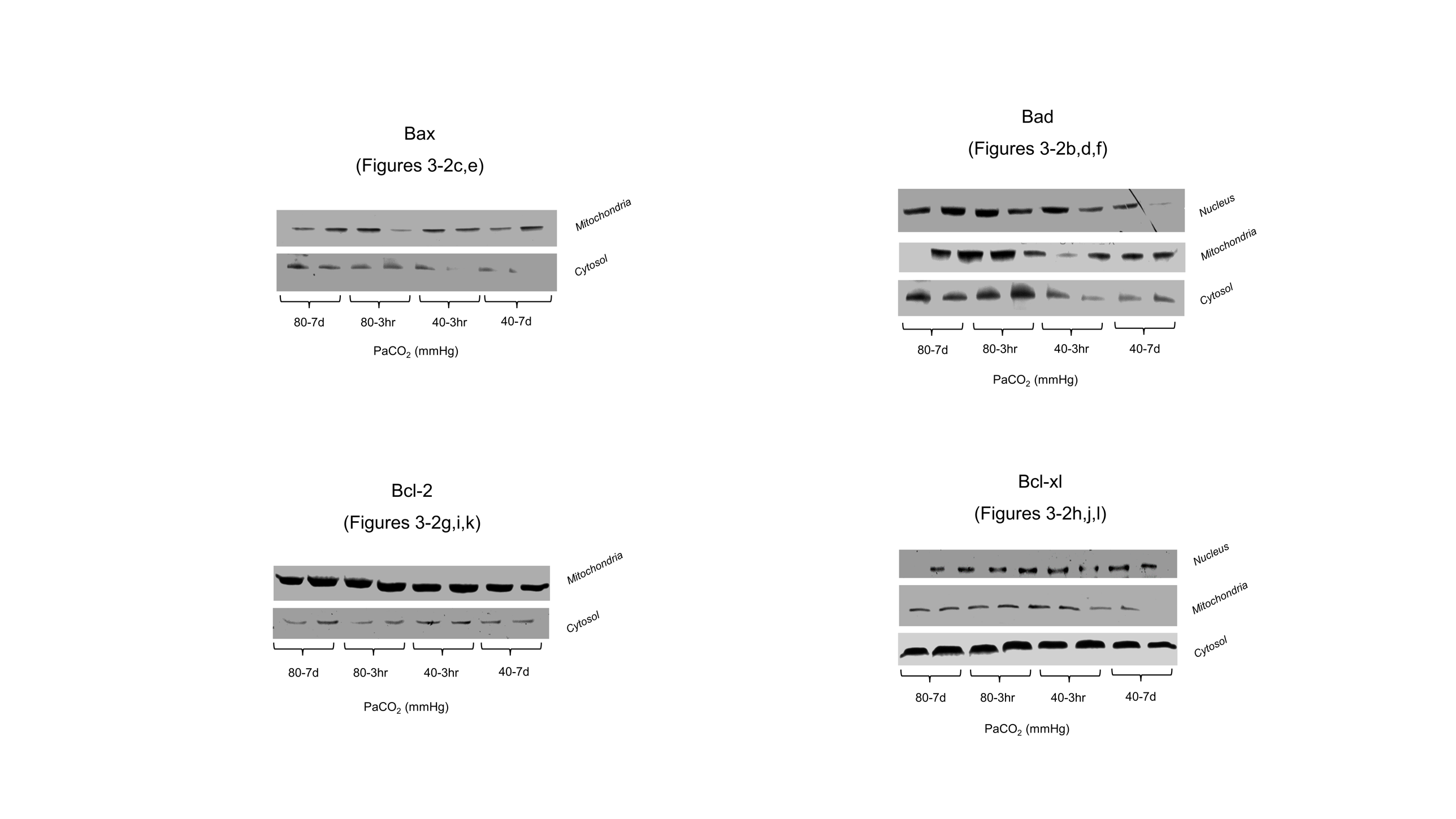

Supplement: Extended Data Fig 3-3 — Shows representative western blots of Bax, Bad, Bcl-2, and Bcl-xl in neuronal nuclei, cytosol, and mitochondria following HC exposure. Download Extended Data Fig 3-3, TIF file. [file eneuro-11-ENEURO.0268-23.2023-s007.tif]

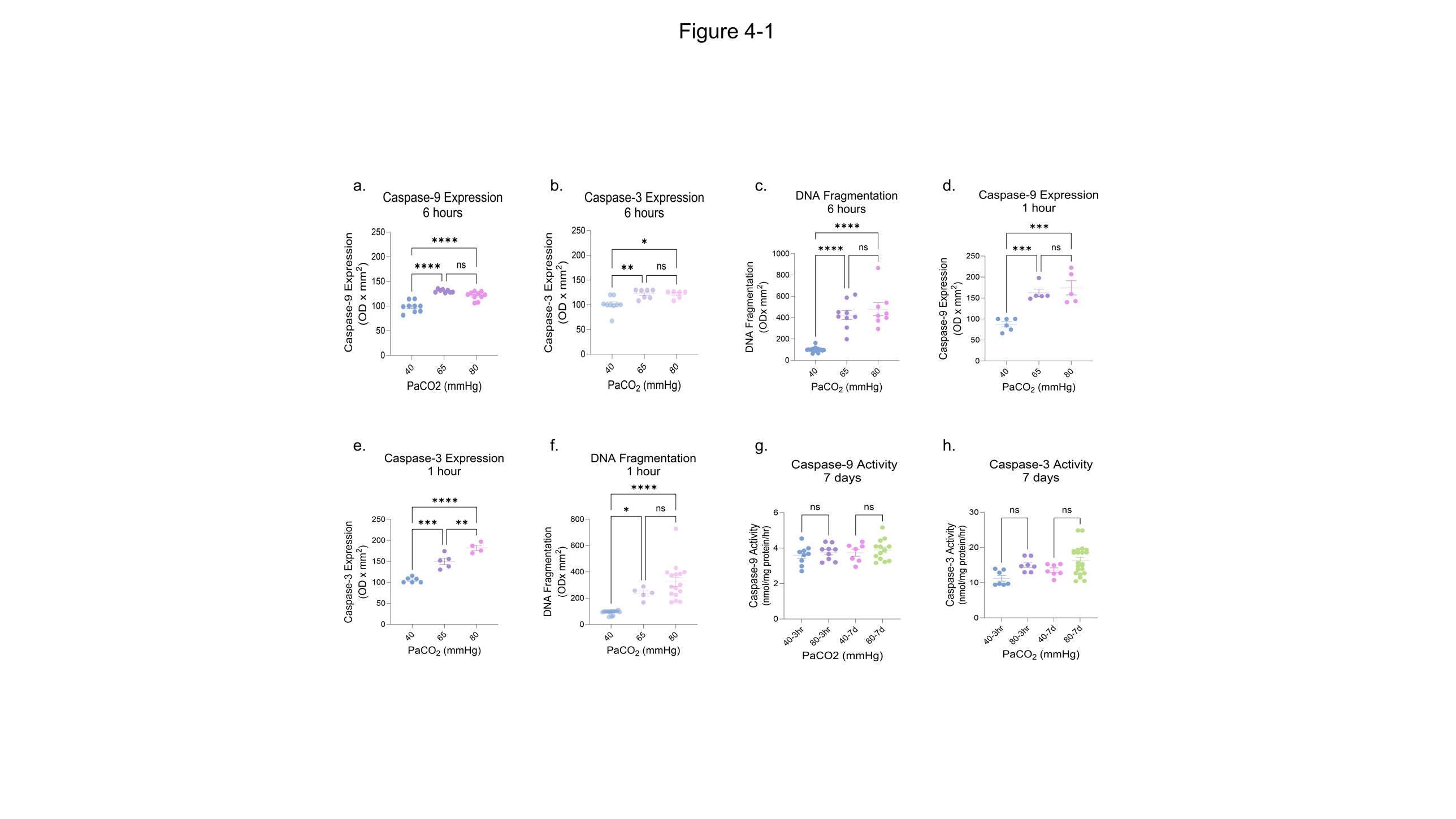

Supplement: Extended Data Fig 4-1 — The effect of HC on of caspase-9 and caspase-3 expression levels and activity and on nuclear DNA fragmentation in cortical neurons showed (a,b,d,e) an increase in caspase-9 and -3 expression following both six hours and one hour of HC, (g-h) but no significant changes in caspase-9 or caspase-3 activity were observed after three hours of HC or following the seven day recovery period. (c,f) There was a significant increase in DNA Fragmentation after one and six hours of HC. Each of the above groups of piglets was exposed to either moderate (PaCO2: 65mmHg) or severe (PaCO2: 80mmHg) HC for one, three or six hours. Each group was compared to a group of NC sham piglets with similar instrumentation and timeline of events. DNA: Deoxyribonucleic acid; HC: Hypercapnia; PaCO2: Partial pressure of carbon dioxide; NC: Normocapnia. Statistical analysis was performed using one-way analysis of variance for multiple groups by Prism statistical software, and the graph displays mean ± SEM values; * p<0.05, ** p<0.01, *** p<0.001, **** p<0.0001; n=5-6/group. Download Extended Data Fig 4-1, TIF file. [file eneuro-11-ENEURO.0268-23.2023-s008.tif]

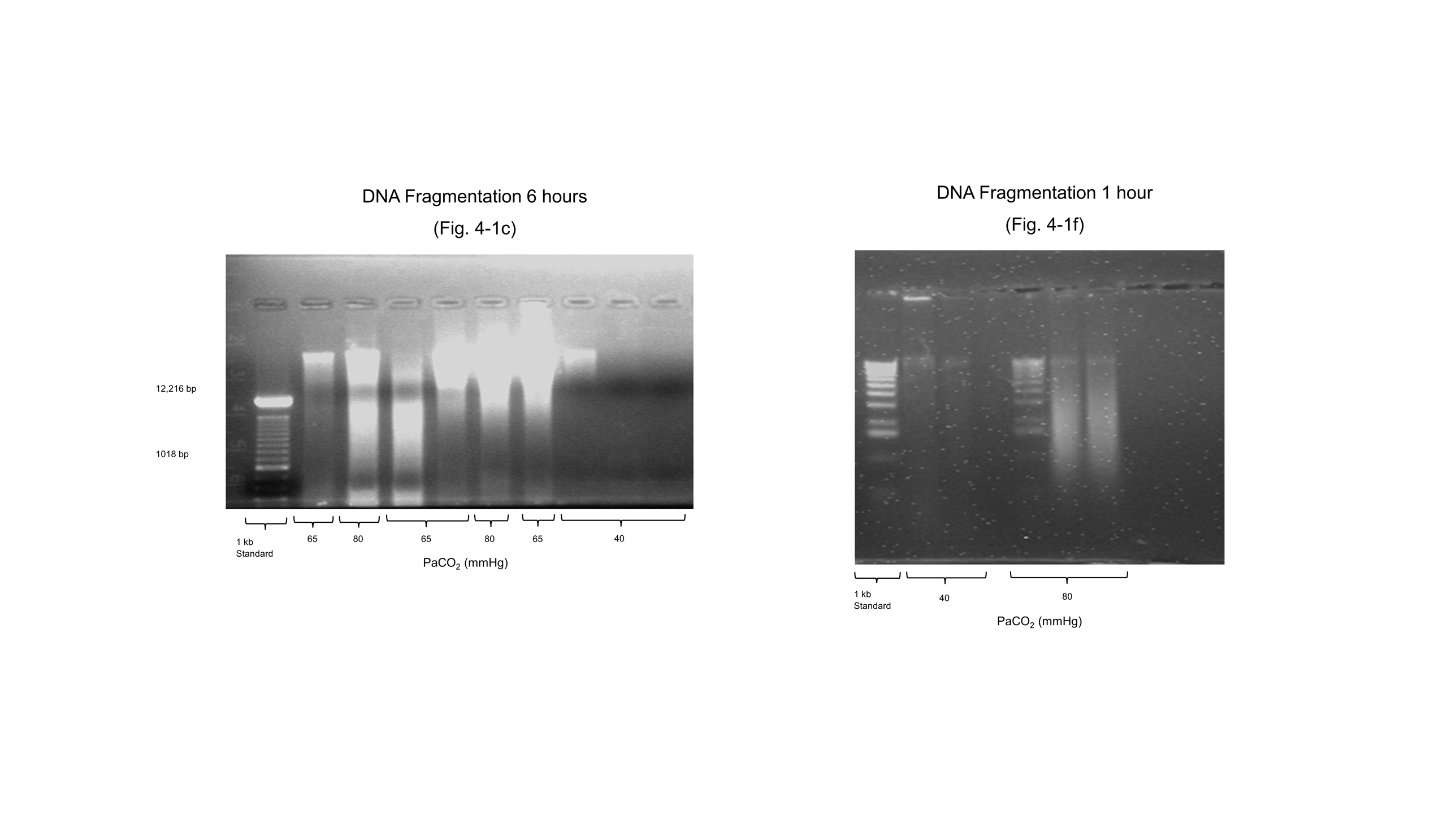

Supplement: Extended Data Fig 4-2 — Shows representative western blots of DNA Fragmentation in 6 hour and 1 hour HC and NC piglets. Download Extended Data Fig 4-2, TIF file. [file eneuro-11-ENEURO.0268-23.2023-s009.tif]

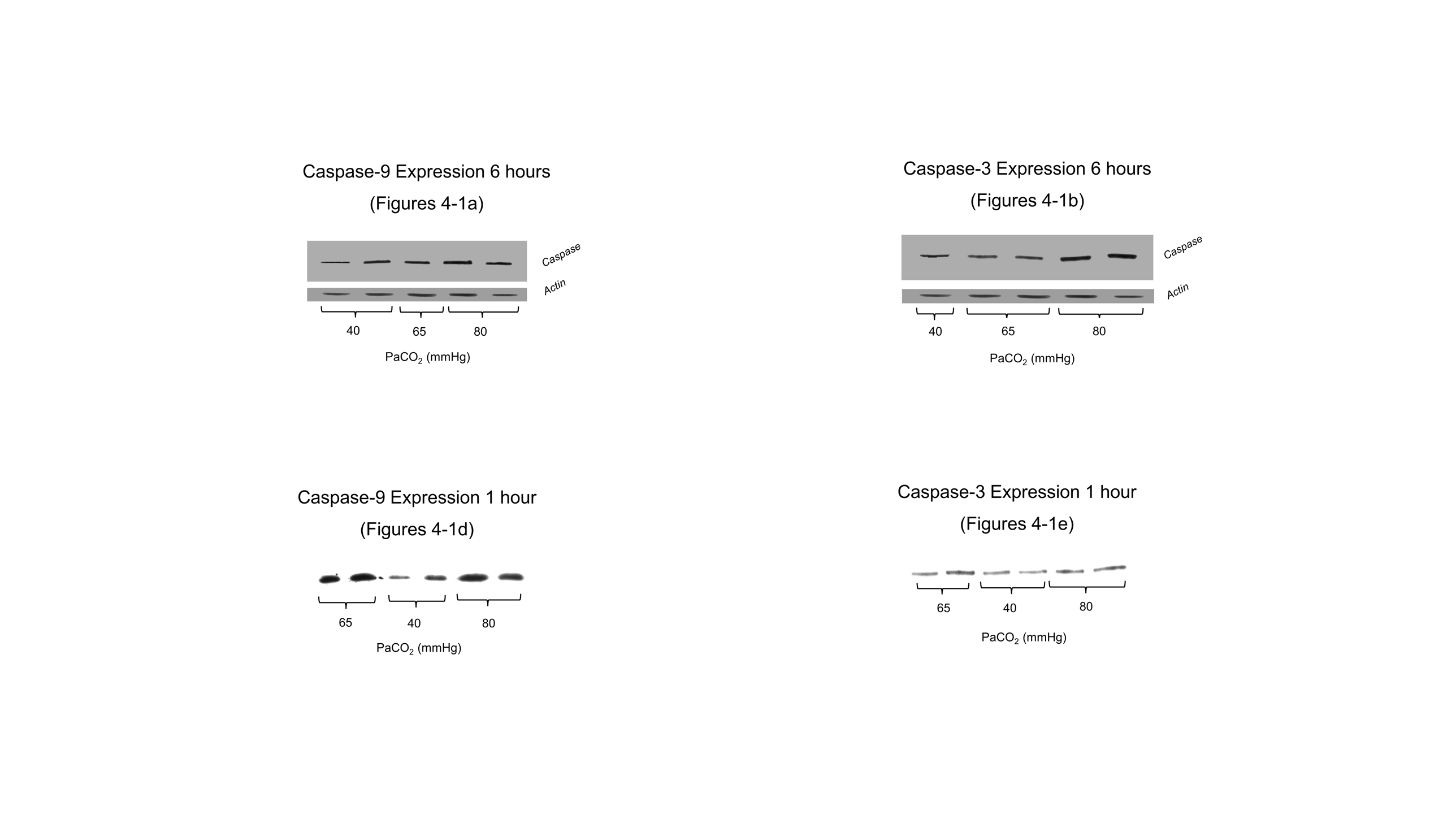

Supplement: Extended Data Fig 4-3 — Demonstrates representative western blots of caspase-9 and caspase-3 expression in 6 hour and 1 hour HC piglets and NC piglets. Download Extended Data Fig 4-3, TIF file. [file eneuro-11-ENEURO.0268-23.2023-s010.tif]

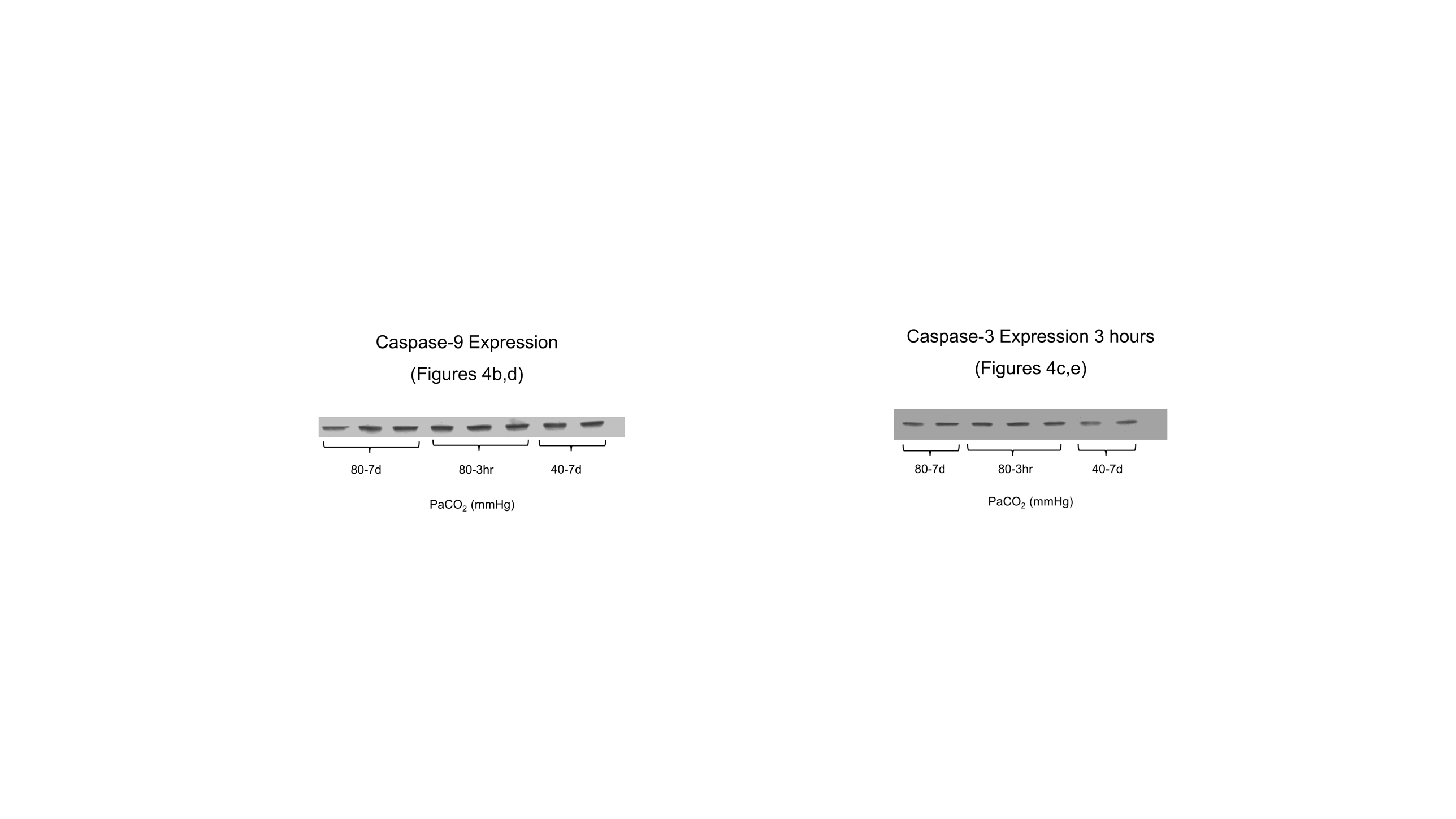

Supplement: Extended Data Fig 4-4 — Shows representative western blots of caspase-9 and caspase-3 expression in recovery HC and NC piglets. Download Extended Data Fig 4-4, TIF file. [file eneuro-11-ENEURO.0268-23.2023-s011.tif]
